# Development and validation of a new methodological platform to measure behavioral, cognitive, and physiological responses to food interventions in real time

**DOI:** 10.3758/s13428-021-01745-9

**Published:** 2022-01-31

**Authors:** M. A. Vargas-Alvarez, H. Al-Sehaim, J. M. Brunstrom, G. Castelnuovo, S. Navas-Carretero, J. A. Martínez, E. Almiron-Roig

**Affiliations:** 1 Center for Nutrition Research, University of Navarra, 31008 Pamplona, Spain; 2 Department of Nutrition, Food Science and Physiology, Faculty of Pharmacy and Nutrition, University of Navarra, Pamplona, Spain; 3 School of Biological and Health Sciences, Technological University Dublin, Dublin, Ireland; 4 School of Psychological Science, University of Bristol, Bristol, UK; 5 Spanish Biomedical Research Centre in Physiopathology of Obesity and Nutrition (CIBERobn), Institute of Health Carlos III, Madrid, Spain; 6 Navarra Institute for Health Research (IdiSNa), Pamplona, Spain

**Keywords:** Portion-control plate, Satiety, Universal Eating Monitor, Automatic Gaze Mapping, Portion size memory

## Abstract

To fully understand the causes and mechanisms involved in overeating and obesity, measures of both cognitive and physiological determinants of eating behavior need to be integrated. Effectively synchronizing behavioral measures such as meal micro-structure (e.g., eating speed), cognitive processing of sensory stimuli, and metabolic parameters, can be complex. However, this step is central to understanding the impact of food interventions on body weight. In this paper, we provide an overview of the existing gaps in eating behavior research and describe the development and validation of a new methodological platform to address some of these issues. As part of a controlled trial, 76 men and women self-served and consumed food from a buffet, using a portion-control plate with visual stimuli for appropriate amounts of main food groups, or a conventional plate, on two different days, in a random order. In both sessions participants completed behavioral and cognitive tests using a novel methodological platform that measured gaze movement (as a proxy for visual attention), eating rate and bite size, memory for portion sizes, subjective appetite and portion-size perceptions. In a sub-sample of women, hormonal secretion in response to the meal was also measured. The novel platform showed a significant improvement in meal micro-structure measures from published data (13 vs. 33% failure rate) and high comparability between an automated gaze mapping protocol vs. manual coding for eye-tracking studies involving an eating test (ICC between methods 0.85; 90% CI 0.74, 0.92). This trial was registered at Clinical Trials.gov with Identifier NCT03610776.

**Supplementary Information:**

The online version contains supplementary material available at 10.3758/s13428-021-01745-9.

## Introduction

Obesity is a global health problem with a complex etiology, in which eating behavior plays a central role (EASO, 2019; World Health Organization, 2020). For example, there is currently good evidence that people tend to eat more and gain weight when exposed to large portion sizes (Hollands et al., 2015; Rolls, 2014; Zlatevska et al., 2014), and that improving portion-control behavior at the time of serving and eating, when preparing meals or when shopping, could help curb the obesity epidemic (Steenhuis & Poelman, 2017; Vermeer et al., 2014). However, despite the application of individual-level strategies and even wider environmental approaches, achieving long-lasting portion control remains a challenge (Almiron-Roig, Forde, et al., 2019a; Rolls et al., 2017). This fact underscores the need for a better understanding of human eating behavior, and in particular, around portion size choice.

### Current methodological issues in eating behavior research with a focus on portion size

Existing methods applied in eating behavior research, especially related to portion size, include approaches to estimate amounts consumed by an individual as part of a dietary assessment; methods to analyze cognitive mechanisms related to portion size behavior; and methods to evaluate the impact of portion size manipulations on portion size behavior (Almiron-Roig et al., 2017).

The recent and rapid development of image capturing technology provides a unique opportunity to apply these methods in studies investigating cognitive processes involved in portion size decisions. Current computerized methods to assess intentions about portion size include the method of constant stimuli and the method of adjustment (Forde et al., 2015). Originally designed to compare the expected satiating properties of foods directly on a calorie-for-calorie basis, these methods are now widely applied in portion size research as they allow sensitive analyses of prospective portion size-related decisions in real time (Brunstrom et al., 2008; Brunstrom & Rogers, 2009). In particular, both methods are sensitive to very small manipulations in portion size (typically 20-kcal increments) and can be used across or within a range of foods, and despite being “virtual”, they are highly predictive of the portions that people self-select and consume in real life (Wilkinson et al., 2012).

Portion-size decisions can be influenced by learning (prior food exposure) and by past experience with a portion-control tool, and this is an area where computerized tools offer great potential (Herman et al., 2015; Robinson, Higgs, et al., 2013b). In particular, sensitive methods are needed to assess memory-related processes such as episodic memory and reconstructive memory of events related to the selection and consumption of meal components that form a balanced meal.

The way in which a meal is consumed (also known as *meal micro-structure*), including eating rate, amount of food loaded at each forkful (bite size), and how fast eating speed decreases towards the end of a meal are key factors associated with altered eating behavior (Robinson, Almiron-Roig, et al., 2014a; Westerterp-Plantenga, 2000).

Appropriate methods are needed to study the actual eating behavior process including portion selection in detail. In addition, behavioral assessments need to be integrated with physiological measures to gain a full understanding of the mechanisms involved in eating behavior. Traditionally, meal micro-structure has been analyzed with stationary eating monitors, such as the Universal Eating Monitor (UEM), which measures the decrease in weight remaining on a plate during an eating episode ( Kissileff, 2000). From these data, eating speed, bite size, meal duration and eating deceleration rate can be calculated, as well as cumulative food intake (the pattern of intake across the meal as a function of meal duration), (Yeomans, 2018). A disadvantage of the UEM is that it requires specific infrastructure and expertise to be operated (Kissileff et al., 1980; Yeomans, 2018) and some versions can be highly susceptible to background vibration. In addition, stationary eating monitors are not always suitable to measure free-living eating behavior (e.g., in environments in which people eat on the go). To mitigate this problem, portable versions have now been developed that comprise food scales connected to mobile devices by Bluetooth. Although they have been validated in field studies, they are not widely available (Ford et al., 2009), which has led researchers to use other strategies such as questionnaires, covert observations, or analysis of video recordings using face-recognition software (Forde et al., 2013; Petty et al., 2013; Woodward et al., 2020). However, these methods tend to be more prone to human error, self-reported eating rate is a poor predictor of objective individual measures especially under free-living conditions (Petty et al., 2013; Woodward et al., 2020), and some methods may be time-consuming, leaving the optimization of the traditional eating monitor (UEM) as a better option.

Finally, to understand the role of visual cues, sensitive measures of visual attention are needed. For example, longer fixation times could explain the formation of visual memories and influence decisions about portion size choice via learning or anchoring processes (Marchiori et al., 2014). Actual visual attention is difficult to measure but can be inferred from gaze movements collected with eye-tracking devices, taking care of the limitations and challenges involved (Orquin & Holmqvist, 2018).

While eye-tracking methodology has been applied previously in food behavior studies (Bollen et al., 2020; van der Laan et al., 2017; Werthmann et al., 2011), very few studies have employed it in the context of actual consumption, with typical setups involving food images rather than real food (Benjamins et al., 2021). The closest study in which this technique was applied with real food in an actual eating event, focused on food observations prior to actual consumption (Wang et al., 2018). Specific challenges of applying eye-tracking to measure food intake behavior include the potential complexity of the stimuli, the dynamic action of eating, including natural head movement and food disappearance from the plate, and the need to control distractions if using natural eating environments. Despite these limitations, eye-tracking can offer a unique insight into cognitive processes related to human eating behavior.

Portable eye-trackers offer a good starting point to examine gaze patterns elicited by real food stimuli but as with other gaze capturing technology, they can generate vast amounts of data. Careful development and piloting of a bespoke image coding protocol is necessary. Particular attention is needed if using automatic gaze mapping (AGM) software, as false-positives may occur if stimuli include adjacent areas of interest (AOI), such as in the case of a composite meal served on a tray or plate. AOIs are used to link eye movement with stimuli or parts of a stimulus, and data analysis derived from AOI statistics, such as dwell time, is used to determine how attention is being directed (Hessels et al., 2016), and how information is being processed (Irwin, 2004).

### Empirical context of this work

In order to gain a full understanding of the causes and mechanisms involved in overeating and obesity, measures of both physiological and cognitive determinants of eating behavior need to be integrated. For example, secretion of certain satiety-inducing gut peptides and appetite hormones seems to be influenced by the sensory properties of food (Lasschuijt et al., 2020; Yeomans et al., 2016). Also, visual feedback from food amounts present on a plate or glass have been shown to impact eating rate and bite size (Almiron-Roig et al., 2015; Wilkinson et al., 2016), confirming that several physiological and psychological processes interact to modulate eating behavior. Despite this, there is a general gap in integrative research in this field because of the inherent difficulties in measuring these parameters simultaneously and in real time. Behavioral and physiological measures tend to interact, and the extent to which this occurs may vary across individuals (Allison & Baskin, 2009) (Crum et al., 2011; Wadhera & Capaldi-Phillips, 2014). This means that combining and synchronizing visual attention parameters, meal micro-structure, and hormonal responses can be complex, however, this step is essential in order to relate behavioral, cognitive, and physiological responses to food interventions in real time.

The present work was developed within the context of a study investigating portion-control mechanisms but has the potential to be useful across a wider range of eating behavior contexts.

How exactly portion-control strategies work is still not fully understood but a series of potential mechanisms have been proposed (Almiron-Roig, Forde, et al., 2019a). These include ways in which food is served or presented (e.g., portion size and packaging cues, size and design of packaging or tableware); ways in which the food is eaten (e.g., speed, distraction); how portion sizes are perceived (e.g., “appropriateness” or norms,); and factors interacting to modulate these perceptions (e.g., palatability). Environmental factors related to cost and labelling/packaging may also play a role (Benton, 2015; English et al., 2015; Herman et al., 2015; Steenhuis & Poelman, 2017).

Knowledge gaps remain, particularly around the cognitive processes involved in long-term (6 months and beyond) portion control and how the effects differ across gender and with body weight. Previous studies suggest that visual cues from food and food containers are able to elicit both physiological and cognitive processes that eventually modulate intake (Almiron-Roig, Forde, et al., 2019a; Wadhera & Capaldi-Phillips, 2014) (Wansink & van Ittersum, 2003). Within this context, the design of tableware has been shown to be a potentially effective strategy to modulate intake by aiding portion control. In particular, by the presence of visual cues such as calibration marks (Vargas-Alvarez et al., 2021).

**Fig. 1:**
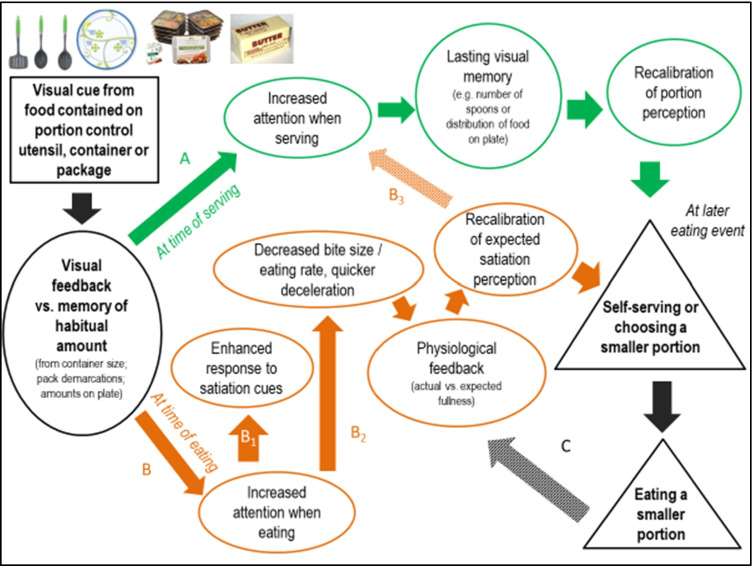
Potential mechanisms involved in portion control based on the effects of visual cues in tableware and food packaging. Two mediator pathways are considered, one at the time of serving (*green arrows* and *circles*); and one at the time of eating (*orange arrows* and *circles*). Stimuli are indicated in *rectangles*, mechanisms in circles and behaviors in *triangles* (see text for literature sources)

Visual cues may impact portion size via changes or renormalization of the so-called *portion-distortion effect*, that is, by reducing the tendency to perceive large amounts as normal (Schwartz & Byrd-Bredbenner, 2006). By influencing portion norms, visual cues are likely one of the strongest potential mechanisms involved in portion control (Marchiori et al., 2014; Robinson et al., 2016; Robinson & Kersbergen, 2018), meal micro-structure (Almiron-Roig et al., 2015) and, indirectly, fullness expectations (Brunstrom & Rogers, 2009). A number of proposed mechanisms and pathways have been identified and these can be represented in a logic model (Fig. 1).

According to this model, foods presented in specialist containers that show volume or portion demarcations; or in plates with demarcations for individual components (e.g., meat and vegetables) of the same meal, provide a visual feedback on the amounts selected or consumed. Alternatively, modified serving/eating utensils and packaging containing portion size demarcations can also provide feedback on amounts served or consumed.

These visual cues also create a contrast vs. the pre-existing memory of a habitual portion for that food. At the time of serving (pathway A in Fig. 1), this visual feedback could increase attention and act as a basis for the generation of lasting visual memories which may then help to update beliefs about what constitutes a normal portion size (Almiron-Roig, Majumdar, et al., 2019b; Haynes et al., 2019; Robinson & Kersbergen, 2018). This may be mediated by anchoring effects, that is, any amount that appears normal in size may guide decisions on how much to self-serve and in some cases may contribute to meal termination (Haynes et al., 2019). However, these effects may be attenuated by repeated exposure to smaller than normal portion sizes over several days (Haynes et al., 2020).

On the other hand, at the time of eating, visual feedback from demarcations on utensils etc. may also increase attention, potentially enhancing the response to internal satiation cues that might otherwise be ignored if eating while distracted (pathway B1 in Fig. 1) (Robinson, Aveyard, et al., 2013a). Visual feedback from container size or pack demarcations or amounts on plate can also influence bite size, eating rate and changes in eating rate (Almiron-Roig et al., 2015; Mishra et al., 2011; Wilkinson et al., 2016) (pathway B2, Fig. 1). Such changes in meal eating behavior may lead to changes in the response to satiation cues and eventually help to recalibrate perceptions about the expected satiation of foods, a factor shown to influence portion choice (Brunstrom, 2011; Brunstrom et al., 2010). Based on these recalibration processes, it is possible that changes at the time of eating can influence future behavior at the time of serving (pathway B3, Fig. 1).

Overall, two processes mediating the impact of portion-size control are proposed: recalibration of portion-size norms and recalibration of expected satiation. Further studies are necessary to confirm these pathways. Nevertheless, approaches focusing on reducing self-served amounts have the potential to reduce overall energy intake without significant compensatory behavior (perhaps via physiological feedback, pathway C) and can therefore help with weight management (Haynes et al., 2020).

### Objectives of the study

To address the existing research and methodological gaps outlined above, we designed an intervention where 76 participants (both lean and with overweight/obesity) self-served and consumed food from a laboratory buffet, using a portion-control (calibrated) plate with visual stimuli for appropriate amounts of main food groups, or a control (conventional) plate in a random order. Volunteers completed behavioral and cognitive tests before and after the meal using a novel combined methodological platform. The platform was specifically designed to allow integrative measures and included a portable eye-tracking device to analyze gaze behavior (as a surrogate for visual attention), an optimized eating monitor that recorded eating speed and related parameters in real time, and software to assess memory for recent portion sizes. At various time points, participants also completed subjective ratings of meal liking and satiety. Portion sizes for all foods chosen and consumed were covertly measured (Robinson et al., 2015; 2014, b), and in a sub-sample of participants, their hormonal satiety response was measured. To assess compensatory eating behavior, participants also completed portion perception questionnaires and a food diary for the remainder of the day.

Recognizing differences in portion size and satiety responses between men and women (Brunstrom et al., 2008; Lewis et al., 2015), the study recruited mainly women, however, a self-selected opportunity sub-sample of men was included to explore the role of visual stimuli on meal micro-structure. The main study hypothesis related to the effects of using a portion-control plate that incorporated a visual guide to appropriate portion-size selection. Specifically, we hypothesized that this ‘calibrated plate’ would increase fixation time (as a proxy for visual attention) on each meal component, resulting in a better control of meal portion size compared with a traditional plate. The main outcome measure was the differences in fixation (dwell) time between the area of interest (AOI) for vegetables, starch or protein in the calibrated plate vs. the control plate. For the sub-study in men, the main outcome measure was the difference in bite size between the calibrated and the control plate. Additional outcome measures included changes in portion size selection and intake for the whole meal and each meal component, 8-h energy compensation, subjective appetite ratings, meal microstructure, memory for portion sizes, portion size norms, portion-size self-efficacy, plate acceptance, meal liking, and biomarkers for the satiety response (women only).

Initial fit-for-purpose confirmation data for the methodological platform and protocol was obtained from the first 20 volunteers completing the study (Vargas et al., 2018). Here, we present the development and further validation of the combined methodological platform using data from the 76 volunteers for the memory test and meal microstructure analysis, blood sample procedure (*n* = 31); and eye-tracking data coding protocols (sub-sample of ten video-recordings).

## Methods

### Study design

The study followed a within-subjects (cross-over) design where participants were randomized to two lunch sessions at our laboratory, one where they ate with the calibrated plate and one with the control plate. After a 7–15 day washout period, participants reversed conditions and repeated the same measures.

Sample size requirements were estimated using an on-line sample-size calculator (http://powerandsamplesize.com/). For a cross-over design, a minimum of 30 women of the same BMI group were necessary to detect minimal differences in fixation (dwell) time of 325 ms per area of interest (AOI) between plates, assuming a SD of 445 ms, 80% power, and alpha 0.05 (van der Laan et al., 2017). Taking into account potential variability in visual attention measures between women with and without overweight, a sample of 60 women was necessary (30 lean and 30 with overweight or obesity). This also covered sample size requirements for analysis of meal micro-structural parameters (*n* = 60) (Laessle et al., 2007) and the gut peptides insulin, ghrelin, and pancreatic polypeptide (*n* = 30) (Yeomans et al., 2016). In addition, a sample of men (all lean) were also recruited to explore meal-microstructural parameters. Based on published data (Laessle et al., 2007), the required minimum sample size for men for this exploratory sub-study was 23, assuming a minimum difference in bite size of 2.4 g between plate conditions (80% power, alpha 0.05, SD 2.9 g). Assuming an expected 12% drop-out rate (Almiron-Roig et al., 2015), the recruitment sample size aimed for was 68 women and 26 men. The study was terminated in March 2020 due to the COVID-19 epidemic, at which point the sample comprised 65 women and 11 men.

Ethical approval for this trial was granted by the University of Navarra Research Ethics Committee on 27 April 2017 and 17 November 2017 (revised version). Informed consent was obtained from all individual participants included in the study. Those completing the study were offered a crockery portion-control plate in compensation for their time and effort. The trial was registered at Clinical Trials.gov with Identifier NCT03610776.

### Participant recruitment

Participants were recruited between September 2018 and February 2020 from Pamplona and surrounding areas via flyers and newsletters, and from an internal database of existing volunteers. To diminish alterations in normal eating behavior due to knowledge of the true study aims (i.e., measuring of portion sizes) (Robinson et al., 2015; 2014, b) the study was advertised as “a study to validate a new plate for healthy eating”. Exclusion criteria included pregnancy/lactation, smoking, performing ≥10 h of intense physical activity per week, alcohol intake >14 units (women) or 21 units (men) per week, vegetarian/vegan; malnutrition, impaired visual or gastrointestinal function; presence of eating disorders (EAT-26 score of ≥19) (Rivas et al., 2010); consuming breakfast < 5 days per week, using a medical device interfering with the eye-tracker (e.g., pacemaker), epilepsy, visual impairment, taking medication affecting vision, appetite or body weight, being on a diet to gain or lose weight and avoiding/disliking the study foods. Participants requiring corrective lenses for eating and who did not use contact lenses were excluded. To be eligible for the study all subjects had to pass a screening and familiarization session prior to the first lunch session where they consumed 125 g of yogurt on the UEM while wearing the eye-tracking glasses and completed electronic questionnaires on the UEM. Participants also tasted and rated the study foods (boiled rice, meatballs in sauce, boiled peas, boiled carrots). Those assigning a score of 40 or more on a 100 mm VAS for the rice, meatballs and at least one of the vegetables, plus producing valid video and UEM outputs, were enrolled.

### Procedures

**Fig. 2:**
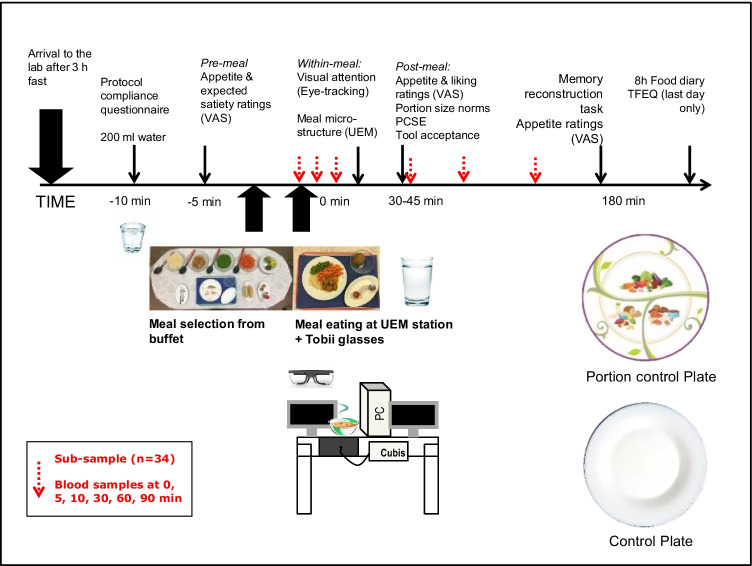
Daily procedure for participants and components of the combined methodological platform. Plates used for this study are depicted on the bottom, right (for details, see text). *Abbreviations*: PCSE, portion-control self-efficacy scale; TFEQ, three-factor eating questionnaire; UEM, universal eating monitor; VAS, visual analogue scale questionnaire. Portion-control plate picture courtesy of Precise Portions LLC, Virginia, USA

The daily procedure for the volunteers is shown in Fig. 2. Participants arrived at the lab after a 3-h fast at a convenient time between 11:30 and 14:30 (starting time was kept constant across sessions). At each session, they completed a ‘protocol check’ questionnaire and consumed 200 ml of still water to standardize thirst levels. Participants were then accompanied to a room where they chose foods from a hot meal buffet without the investigators being present. Participants then moved to the eating station located in a separate room and consumed the meal on their own, while wearing a portable eye-tracking device (Tobii ProGlasses 2, Stockholm, Sweden). The eye-tracker was fitted and calibrated before the meal was brought in. Immediately before and after eating, and 180 min after the meal, participants completed subjective satiety ratings on a computer screen in the eating station. Participants were then allowed to leave the lab for 3 h but could not consume any food or liquid except for non-carbonated water. Over this period, activities were not monitored but participants were asked to maintain the same physical activity and daily routines across sessions. At 180 min, participants returned to the lab to undertake a computerized image-based test that assessed their memory for the portion sizes consumed previously. Before leaving, participants were instructed on how to complete an 8-h food record and were given the record to fill in at home and return on the next session. A sub-sample of participants provided blood samples immediately before, and 5, 10, 30, 60, and 90 min after the meal.

The protocol was developed to minimize overlapping measures from the various techniques as much as possible. To decrease confounding due to protocol demands, participants providing blood samples were instructed to look away from the plate and preferably pause eating while the nurse carried out the blood draws and the lapse of blood extractions was subtracted from the overall meal time. Video data during extraction were also excluded from analyses.

The study variables and corresponding measuring instruments were:

- *Demographic and anthropometric variables* (obtained using published questionnaires and for weight/height, direct measuring in the laboratory): age, sex, weight (kg), height (cm), body mass index (BMI) in kg/m^2^, weekly hours of moderate to vigorous physical activity; consumption habits and household composition.
- *Behavioral variables*: Eating behavior traits, measured with the EAT-26 and TFEQ questionnaires (validated versions for the Spanish population) (Garner et al., 1982; Rivas et al., 2010; Sánchez-Carracedo et al., 1999; Stunkard & Messick, 1985); Habitual portion size (portion size norms), liking, and expected satiety for the meal, measured using published 100 mm VAS (Forde et al., 2015, Robinson et al., 2016); Hunger, fullness, thirst, nausea before, immediately after and at 3 h post meal, measured with validated 100 mm VAS (Hill & Blundell, 1982); Portion-control self-efficacy, assessed through the validated PCSE scale (five-point Likert scale ranging 1–40 points) (Fast et al., 2015), translated into Spanish and back-translated by a professional translator, plus verified with the authors (Jennifer Harman, personal communication); Portion tool acceptance, measured with a shortened version of a piloted questionnaire (five-point Likert scales) (Almiron-Roig et al., 2016), translated into Spanish and back-translated by a professional translator, plus verified with the authors; Meal micro-structure measured with the Universal Eating Monitor (UEM) (Yeomans, 2000), including meal duration (min), eating rate (g/min), bite size (g), and deceleration rate (g/s^2^); Gaze dwell time (in ms) (Werthmann et al., 2011) for main areas of interest (AOIs) of the meal, measured with the Tobii ProGlasses 2; Energy compensation (adjustment) at the end of the day (using data from an estimated food diary) (Almiron-Roig et al., 2013) (see Supplementary information for further details).
- *Metabolic parameters*: blood glucose, insulin, pancreatic polypeptide and ghrelin in serum and plasma before and at 5, 10, 60, and 90 min post-consumption (Yeomans et al., 2016). The hexokinase test (Horiba ABX, Montpellier, France) was used for blood glucose and enzyme-linked immunoassay kits were used for insulin (Mercodia, Uppsala, Sweden), total ghrelin (Merck KGaA, Darmstadt, Germany) and pancreatic polypeptide (Millipore, Missouri, USA), following manufacturer’s instructions. For ghrelin analyses, a protease inhibitor (Pefabloc, Sigma-Aldrich), was added before processing the samples to reach a final concentration of 1 mg/ml. Once centrifuged, hydrochloric acid (final concentration 0.05 N) was added to the plasma before storage at -80°C.
- *Cognitive variables*: episodic memory for eaten portions analyzed at 3 h post consumption through a computerized task using a bespoke computer program (Brunstrom, 2014).

### Study plates

The calibrated plate included printed demarcations and illustrations (portion size guidelines) for recommended amounts of protein foods, starchy foods, and vegetables based on US Department of Agriculture guidelines. It was specifically designed for this study by Precise Portions NLS based on previous research (Almiron-Roig et al., 2016; Almiron-Roig et al., 2019b). The control plate was a white dish of the same size and depth but slightly lighter in weight; it was purchased from Group Carrefour, France. Both were ceramic plates, microwave and dishwasher safe, with an enamel finish. Both plates measured 25 cm in diameter including a 3.5-cm rim (Fig. 2 and Supplementary Figs. S1, S2).

### Study foods

The buffet included popular foods consumed in Spain as part of a main meal and were: seasoned white rice (Brillante, Sevilla, Spain), boiled peas and boiled carrots (brand Carrefour, France), meatballs in sauce (brand Carrefour), olive oil (Capricho Andaluz, Córdoba, Spain), wholemeal bread, salt and pepper (brand Carrefour) (Supplementary Fig. S1a). The oil was presented as 10-g individual servings. The bread was made on the day in a local bakery and presented in two 50–60 g portions (rolls). The rice, vegetables, and meatballs were heated to 66°C and were offered in 400-g portions that were served in transparent bowls with identical serving spoons. These four foods were chosen because they match the nutritional composition required when using the calibrated plate and because no cutting is required, which obviates the need to apply force to the balance in the UEM (which would alter the readings). Due to their large size, meatballs were presented halved. Complimentary fruit and water were provided after the meal. Foods depicted for the memory reconstruction test were exactly the same foods excluding the bread, fruit and condiments (which were optional). Foods were heated and presented in the same exact format as in the meal, before being photographed.

### Equipment

#### Optimized Universal Eating Monitor (UEM)

**Fig. 3:**
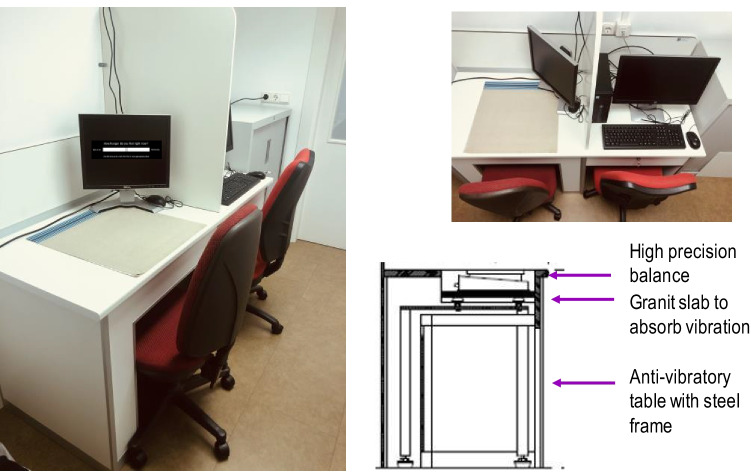
Universal Eating Monitor station at the University of Navarra. *Left and upper right,* room setting displaying the function for visual analogue scale (VAS) questionnaire on one of the screen monitors. *Bottom right,* lateral view outline of the anti-vibratory table hosting the high precision balance (Diagram courtesy of Borda Laboratorios, Madrid, Spain)

The UEM is designed to analyze meal micro-structural parameters (i.e., bite size, eating rate, deceleration rate, and meal duration) when a volunteer consumes a meal sitting at a table (Yeomans, 2000). An optimized UEM station was designed and built in-house for this study with particular attention to minimizing background vibration. It was placed in an isolated testing room within the Nutrition Interventions Unit with only artificial lighting from above and constant temperature below 30°C. The UEM components comprise a concealed precision scale (Sartorius Model MSA5201S-1CE-D0), a PC, and two screen monitors. The scale is connected with a serial line to the PC and is located beneath a purpose-built table under a hole, on top of which a placemat is secured to allow positioning of the plate. The UEM at the University of Navarra has been purposely built on a bespoke anti-vibration table containing a steel frame and granite slab measuring 2 x 35 x 35 cm, upon which the Sartorious balance rests. The balance includes an auto-calibration function and electronic adjustable levelling legs. It also carries a detachable digital viewer located in a lockable, adjacent drawer (Fig. 3).

The PC hosts the Sussex Ingestive Pattern Monitor software (SIPM) (Yeomans, 2000) supplied by the University of Sussex (U.K.) and programmed to record weight readings from the scale at 2-s intervals (precision 0.1 g). From these readings, the average bite size (the difference between each two consecutive weight records), eating rate (grams consumed per minute), and deceleration rate (grams consumed per squared second) are calculated. A dual screen system is used to allow the investigator to program the software away from the volunteer´s view using a dividing panel.

The volunteer´s screen displays electronic questionnaires and step-by-step instructions for the participant.

#### Eye-tracker

The Tobii Pro Glasses 2 is an eye-tracking device that is designed to measure gaze movement and pupil dilation during exposure to 3D stimuli. It comprises a wearable head unit (glasses), a recording unit, and controller software. The head unit detects eye movement and point of gaze at a frequency of 50 MHz, directed to any 3D space using near-infrared illumination from four eye cameras. It also accommodates a front-viewing (‘world’) camera that can record the direction of gaze across multiple items in a visual scene (Supplementary Fig. S1b). The glasses are connected to a portable recording unit from which video output can be retrieved and downloaded to calculate dwell times on AOIs. The recording unit is connected to the head unit via an HDMI cable and it stores the data on an SD memory card. It is controlled from a tablet or computer running the controller software on a Windows operating system, facilitating the managing of participants, controlling the eye-tracker, and viewing both real-time and recorded eye tracking data. Calibration is performed automatically with the controller software prior to each participant’s test (1-point calibration system). The average accuracy for the Tobii Pro Glasses 2 lies around 1.42 ± 0.58 ° and the average precision around 0.34 ± 0.16 ° (MacInnes et al., 2018).

#### Memory reconstruction software

**Fig. 4:**
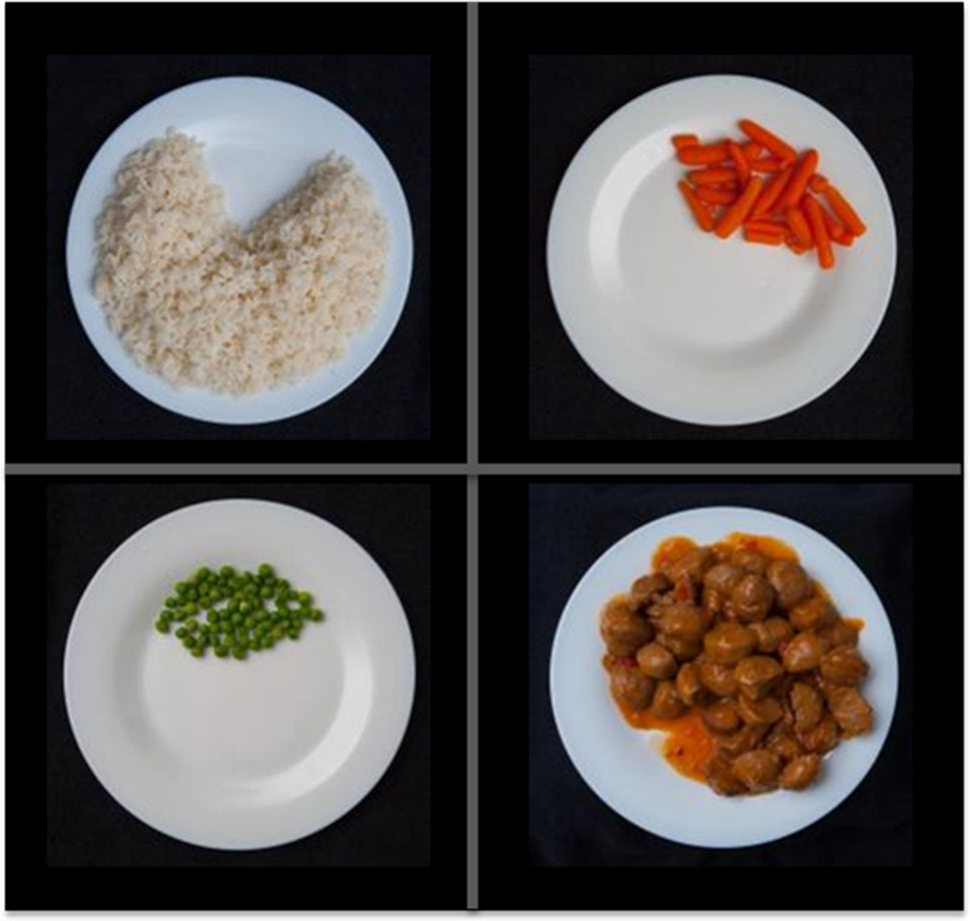
Screenshot of the portion-size reconstruction software. The food photos were taken by a professional photographer using a digital camera with constant lightning and angle, and the same control dish. Portion sizes started at the equivalent of 1 tablespoon and followed by 20 kcal increments until the food filled about 80% of the plate (assumed to be the maximum volume physically fitting in the plate, based on the study protocol, which required selecting at least three meal components). The initial 1 tablespoon portion is based on 50% of the average small portion of cooked rice for Spanish consumers (Russolillo & Marques, 2008). The 20 kcal increments are based on previous research using full plates (Brunstrom, 2014). For carrots, due to their low energy density, 5 kcal increments were used instead

Bespoke software was developed for this study to measure episodic memory for portion sizes chosen/consumed in a previous eating occasion. It was designed by the Nutrition and Behaviour Unit at the University of Bristol (U.K.) and was coded using Visual Basic. The software enables participants to change the size of a portion of food on a computer screen. In this specific version, sub-components of a meal could be selected independently. Images for the starch, protein, and vegetable foods came from a digital photographic atlas generated at the University of Navarra and featured 110 images of the study foods in increasing portion size (starting at 1 tablespoon with 5–20 kcal increments until the maximum feasible volume to fill about 80% of the plate). All images were taken at 90 degrees angle with a Canon EOS 1D Mark III digital camera fitted with an EF 24-70 mm f/2.8L USM (34 mm) lens. The brightness of the raw images was adjusted with Adobe Lightroom (Adobe, California, USA). JPG images were then uploaded to the software, and users adjusted portions of each meal component to generate a personalized virtual plate, based on the method of adjustment (Brunstrom, 2014) (Fig. 4). These data were used to calculate the percentage accuracy in recalled portion sizes of each meal component vs. actual amounts served on the plate (see *Setting up of combined platforms and protocols* below for details of the actual task given to participants).

### Setting up of combined platform and protocols

#### Software installation

Programming of the SIPM software (UEM) for the screening and intervention sessions was carried out according to the manufacturer instructions in a way that enabled measures to be synchronized with eye-tracking video recordings. For both sessions, the SIPM featured an initial appetite questionnaire followed by the food intake test. The appetite questionnaire included randomly ordered 100-mm VAS questions for hunger, fullness, thirst, and nausea. This questionnaire was displayed just before and just after the intervention meal to measure appetite/satiety after eating, in relation to baseline levels. In addition, two additional questions were displayed, one before (100 mm VAS for expected satiety) and one after the meal (100 mm VAS for liking), to collect information about food preference and portion size perceptions (see *Procedures* for references to measuring instruments).

The Tobii eye-tracker was set up following the manufacturer´s instructions in the same environment-controlled room as the UEM. The Pro Glasses controller software was run either from the same PC as the SIPM software or from a separate laptop to avoid software incompatibility. Video data were recorded in MP4 format at 25 frames per second (fps) and stored on SD cards.

The memory software was installed in the same PC as the SIPM software. The same appetite VAS questionnaire featured in the SIPM was included in the memory software as a means to collect appetite/satiety data at 3 h post-meal.

#### Protocol verification for the UEM, eye-tracker and memory test

The UEM protocol requires that participants sit in an upright position without applying any force to the table (from either above or beneath the surface). It also requires that they do not move the food container, that they place cutlery on a side dish after finishing or during meal pauses, and that they do not use mobile or other electronic devices, except when instructed to do so by the investigator (Almiron-Roig et al., 2015). An A4 poster with an info-graphic to remind participants of these requirements was placed in the UEM eating station and participants received verbal instructions just before the start of the test.

The eye-tracker test involves initial calibration and a gaze recording test. Participants received a reminder that the eating session (i.e., only what they saw through the glasses) would be recorded and that they needed to follow the specific instructions for meal eating in addition to instructions for the video recording. Before starting, the glasses were fitted to the volunteer in a way that minimized gazes outside the lenses (i.e., maximizing gazes directed at the food), while ensuring the volunteer felt comfortable enough to eat while wearing them. The volunteers were then instructed to avoid mixing the different foods on the plate and to follow the postural and other requirements specified above for the UEM, plus to look through, rather than above or below the glasses, as much as possible. Volunteers were not made aware that food weight would be recorded to avoid conscious or unconscious alteration of their eating behavior (Robinson et al., 2015; [Bibr CR65][Bibr CR66]). We strived for the plate to always be at the same distance and visual angle to the volunteer, and for its orientation to remain constant (i.e., with vegetables on the top half of the plate). Time for consumption was unrestricted. A bell was provided to participants to alert the investigators once they had finished the food on their plate and any additional bread. Complimentary fruit and water were served after the video-recording and the food intake measures had been stopped.

To avoid priming participants to think about portion sizes, the portion-size memory test was referred to merely as an ‘image test’, that would be completed on a computer 3 h after the meal. At the beginning of the test, volunteers were first shown the physical plate and reminded that it was one of the plates used in the study. They were then asked to complete the appetite questionnaire, and the software then prompted them to “create the portion you consumed at your last meal”. They did this by selecting appropriate portions on the screen, one meal component at a time (Supplementary Fig. S3).

Initial performance of the UEM, eye-tracker and memory software was checked during a mock session and adjustments to the protocol were applied as necessary. The refined combined procedures were administered subsequently to all 76 volunteers. The same standardized instructions were applied in the screening and intervention sessions throughout the study. The quality of the three methods was then evaluated by calculating the number of invalid outputs across the 76 volunteers and across the two sessions. Gaze quality of the eye-tracker outputs and completeness of the UEM outputs were also explored. For the memory software, we compared the recalled and actual portion sizes after food consumption using each plate in a sub-sample of 20 participants.

#### Protocol testing for blood extractions

The feasibility of the blood extraction protocol was initially tested in the first ten volunteers by looking at whether the target timings for blood extractions were compatible with the UEM and eye-tracking measures. Adjustments to the nurse timings and instructions to participants were made as necessary. After obtaining the complete dataset for the 31 volunteers who provided blood samples, we calculated the total blood extraction time employed by the nurse at each session and contrasted real extraction times against target times. Finally, a sensitivity analysis was carried out to isolate any potential effects of the blood sampling procedure on UEM measures (see under Data management & data analysis).

### Development of the eye-tracking image coding manual protocol

For the analysis of video data a bespoke manual coding protocol was first developed in the open-source software Lightworks 14.0 (LWKS Software Ltd, Swindon, United Kingdom), based on standard eye-tracking methodology (Duchowski, 2017; Holmqvist & Andersson, 2017) and previous research (Castellanos et al., 2009; van der Laan et al., 2017; Werthmann et al., 2011). This protocol was piloted using a sub-sample of six recordings and subsequently tested for reproducibility across two independent raters. After applying improvements, it was then used as the basis for the development of a protocol for automatic gaze mapping (AGM) using the software Tobii Pro Lab (Tobii Pro AB, Stockholm, Sweden). Finally, the AGM version was validated against the manual version in a sample of ten recordings, for its future application to the complete sample.

**Fig. 5:**
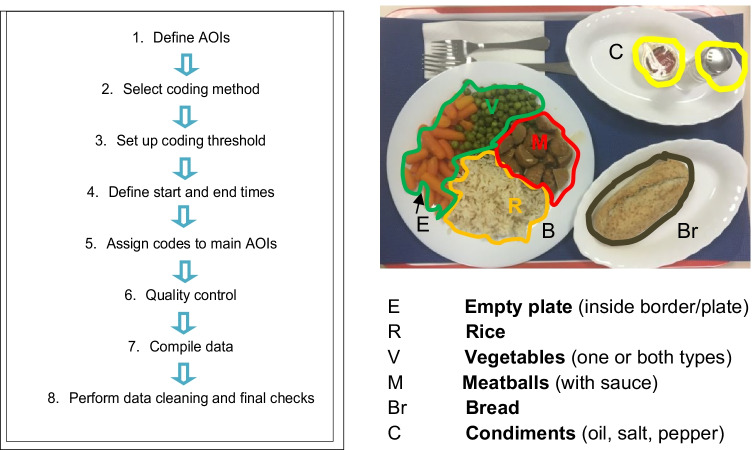
Development of the video data coding protocol with Lightworks 14.0. *Left*, steps followed for the protocol development. *Right,* areas of interest (AOI) used for the initial gaze data analysis. Dwell time on mixed food areas (including more than one AOI) and time on food loaded onto the fork was also initially included

Protocol development in Lightworks was carried out in eight steps, based on current literature (Duchowski, 2017; Orquin et al., 2016; Salvucci & Goldberg, 2000; Werthmann et al., 2011) (Fig. 5). A step-by-step description can be found in the Supplementary Information. Briefly, the process started with defining the areas of interest for the study (AOIs), the coding parameters plus start and end times. This was followed by the assignment of a code to each AOI. Due to the particular nature of the stimuli (food disappearing from a plate), this step necessitated the development of a visual guide to help assign codes in a standardized way (we used the proportion of each food included in the circular marker for the Tobii ProGlasses gaze point as defining criteria). Once all the relevant data had been coded and compiled, quality assessment and data cleaning were performed and the data were explored for plausibility.

### Piloting and reliability of the eye-tracking image coding manual protocol

Several rounds of testing were applied to the initial version of the coding protocol developed in Lightworks to identify challenges and introduce improvements, before validation. These included pilot testing of six representative recordings corresponding to one woman with and another without overweight; and one lean man consuming lunch on both plates.

For the inter-rater reliability (IRR) test, two trained raters independently coded the same two recordings in Lightworks (corresponding to the same lean woman, eating with the two plates). This generated a total of 28 AOIs (14 for each rater) including 29,744 frames. The 14 AOIs corresponded to the seven AOIs examined for each plate, i.e., rice, vegetables, meatballs, border of plate, empty zone, mixed zone, and bread. Percent fixation times for each AOI were calculated by dividing the fixation time (in s) of each AOI by the sum of fixation times for all AOIs, checked for normality and subsequently analyzed with Pearson´s correlation. In addition, we calculated the intra-class correlation coefficient (ICC) and the Reliable Change Index (RCI) (Jacobson & Truax, 1991). The ICC takes into consideration both the between-subjects standard deviation (SD) and the within-subjects SD, and therefore accounts for inter-rater bias (Liu et al., 2016). The RCI (or RC score) identifies pairs of values that do not agree between the two raters (RCI ≥ 1.96 indicates a significant difference between two values).

### Adaptation and validation of the eye-tracking image coding manual protocol to Automatic Gaze Mapping (AGM)

Manual coding of video data, despite allowing high precision, is extremely time consuming and unfeasible for large amounts of data. Thus, a coding protocol allowing automatic gaze mapping (AGM) in Tobii Pro Lab was created by adapting the final Lightworks coding protocol with the aim of analyzing the complete sample of recordings (*n* = 109 after excluding missing sessions and recordings with < 80% gaze capture).

The AGM protocol included the same coding criteria as Lightworks for start and end times, AOIs and metrics, with some adjustments. The default *Tobii I-VT* gaze filter was applied to identify fixations in the desired AOIs. AOIs were initially defined using the in-built *AOI Tool* function in Tobii Pro Lab, which allows hand-drawing, and a screen capture of the image corresponding to the start coding point was used for feature matching. The final AOIs used were the rice, vegetables (all), meatballs and border of the plate. To account for the dynamic nature of the AOIs during the eating episode, we applied adjustments to these AOIs every 10 s, using the function *Select/Move vertices*.

In order to test the validity of the AGM protocol, adjustments were applied to the Lightworks protocol to make it compatible with AGM. Thus, mixed zones were re-coded in Lightworks as a single AOI, representing the most likely fixation point in the context of the specific foods that were being looked at; for gazes including the fork, these were recoded as the food AOI that was present behind the fork, irrespective of the fork being loaded or empty. These adjustments were necessary as it was not possible to code foods loaded on the fork or mixtures of foods in a consistent way using AGM.

The AGM protocol was validated vs. the manual (Lightworks) protocol in a sample of ten representative videos. For each video, we applied the AGM protocol to the first 60 s after starting the meal, with 10-s interval AOI adjustments, and compared the mean dwell time to that obtained with the Lightworks protocol (see *Data management and data analysis*). The recordings were chosen to cover a representative sample of individuals from our study and included a normal-weight woman, a woman with overweight, a woman with obesity and a normal-weight man, eating with both plates. To simplify analyses, we selected subjects not providing blood samples. We selected the first minute at the start of the meal because we observed volunteers move their head less and the AOIs change less dramatically during this period, compared with halfway or the end of the meal. In addition, for the purpose of the study, the first minute is when we expect the visual stimuli to have stronger impact on the satiety responses and memory for portion sizes. Troubleshooting was applied to sort problems related to peripheral view, interference (e.g., bread, fork, and condiments), false-positives due to adjacent AOIs and false-positives on the border AOI.

### Data management and data analysis

Data analyses were carried out in the available dataset from 76 subjects, except when otherwise stated. Variable normality was tested for with the Shapiro–Wilk test. Statistical analyses were conducted in STATA v.12 (StataCorp LLC, Texas, USA). Significance was set at the 0.05 level.

For the UEM and memory software performance, we calculated the probability of invalid outputs (e.g., erroneous or absent data) for each device out of the total available outputs (*n* = 152 for the UEM and *n* = 148 for the memory test).

Mean recalled vs. consumed portion-size differences for each food within and between plate conditions were compared using paired samples *t* tests (or Wilcoxon signed-rank tests for non-normally distributed variables).

For the blood extraction protocol, for the 31 women providing blood samples, we calculated the time required for blood extractions while participants were eating. Two averages were computed, i) a mean ‘absolute duration’ of each blood draw and ii) a mean ‘proportional duration’ - based on the ratio of the duration of the extractions and the total meal duration (*n* = 100 extractions). We also calculated the mean ± 95% CI time difference between target and real times for blood extractions across all times and conditions, as target time minus real time (*n* = 295 extractions). Mean target vs. actual blood extraction time differences between plate conditions were compared using a Wilcoxon signed-rank test.

Potential effects of the blood sampling procedure on UEM measures were explored using independent-samples *t* tests (or Mann–Whitney tests when data were not normally distributed) that assessed differences in mean eating rate, bite size, meal duration, and deceleration rate across women who did (*n* = 31) and did not provide blood samples (*n* = 34).

Eye-tracking data quality was estimated from the percentage of gaze capture (gaze samples) provided by the Tobii controller software across all video recordings (*n* = 149 recordings). A lower threshold at 80% was adopted based on the literature and taking into account the within-subjects nature of the study (Hvelplund, 2014).

Inter-rater reliability of the Lightworks protocol was examined with a three-pronged approach. First, Pearson´s correlation was conducted to determine the strength of the association between rater 1 and rater 2’s scores. Second, the intra-class correlation (ICC) was calculated using an online-excel calculator (Hopkins, 2009) based on published formulas by Bartko (Bartko, 1966) and by McGraw and Wong (McGraw & Wong, 1996):

1 $$\mathbf{ICC}=\left({{\boldsymbol{SD}}_{\boldsymbol{b}}}^{\mathbf{2}}-{{\boldsymbol{SD}}_{\boldsymbol{w}}}^{\mathbf{2}}\right)\div {{\boldsymbol{SD}}_{\boldsymbol{b}}}^{\mathbf{2}}$$

*SD between* subjects (SD_b_) and *SD within* subjects (SD_w_) were calculated for the paired data including 14 AOIs using the Sum of Squares (SS) method and dividing each SS by its degrees of freedom (*df*). For the SD_b_: **df = k – 1.** For the SD_w_: **df = N –fk,** where k is the number of groups and N is the total number of cases for all groups combined. For this study, k = 14 and *N* = 28. For the *between-subjects* calculations the mean of all the AOIs for each rater was used, while for the *within-subjects* calculations the mean of the two raters were used for each AOI (see Supplementary Information for full calculations).

Third, the Reliable Change Index (RCI) was computed using the formula of Jacobson and Truax (Jacobson & Truax, 1991), that is:

2 $$\mathbf{RCI}=\left({\boldsymbol{X}}_{\mathbf{2}}-{\boldsymbol{X}}_{\mathbf{1}}\right)\div \mathbf{Sdiff}$$

where x_1_ is rater 1’s rating and x_2_ rater 2’s rating; and Sdiff is the standard error of the difference between the two measures, calculated as:

3 $$\mathbf{Sdiff}=\sqrt{\mathbf{2}\left({\mathbf{SEm}}^{\mathbf{2}}\right)}$$

SEm is the standard error of the measure, calculated as:

4 $$\mathbf{SEm}=\mathbf{SD}\times \sqrt{\mathbf{1}-\mathbf{ICC}}$$

where SD is the standard deviation and ICC the intra-class correlation. SD can be either the SD of a reference method in a representative population or computed from the sample. For this study, we used the total SD computed from the sample and verified it using one-way ANOVA in STATA. RCI values are standardized *z*-values, therefore an RCI ≥ 1.96 indicates a difference at a significance level of α = 0.05 between any paired values being compared.

We intended to perform Bland–Altman plots as a test of agreement between raters. This analysis assumes normality of the differences between raters. If normality exists, the limits of agreement (range of differences that lay within the mean difference ±1.96*SD) will represent the interval in which 95% of the observations will fall (although not representing acceptable values necessarily) (Bland & Altman, 1986). In this study, the differences between raters were not found to be normally distributed therefore, the limits of agreement were not applicable.

For the AGM protocol validation, mean fixation times per AOI between methods (AGM in Tobii Pro Lab vs. manual coding in Lightworks) were compared across a sub-sample of ten recordings (first 60 s), using paired samples *t* tests. Further, the ICC was calculated as explained above (Bartko, 1966; McGraw & Wong, 1996). Bland–Altman plots were used to explore agreement between methods (Bland & Altman, 1986).

## Results

### Subjects

A total of 76 volunteers (65 women and 11 men) were recruited of which 63 women and ten men completed both lunch sessions (three subjects could not complete the second session due to personal reasons and closure of facilities due to the pandemic lockdown). Amongst the 65 women, 31 were lean/normal-weight and 34 had overweight or obesity. All the 11 men were lean. Subjects had a mean (±SD) age of 41.3 ± 12.3 years, with body mass index (BMI) of 26.0 ± 4.0 kg/m^2^. Thirty-one of the 65 women provided blood samples. Thirty-seven subjects used the calibrated plate first and 39 used the control plate first. EAT-26 and TFEQ scores confirmed no presence of eating disorders.

### Universal Eating Monitor (UEM) performance

Of the 76 volunteers, three failed to attend the second visit, resulting in 149 recovered UEM outputs by the end of the trial (130 valid ones). Of the 130 valid outputs, 63 corresponded to the calibrated plate and 67 to the control plate (66 for visit 1 and 64 for visit 2). Twelve outputs of the original 149 did not contain valid data due to a technical problem with the SIPM software (8% failure rate). Another six outputs had to be excluded because the volunteer did not follow the protocol (on four occasions the volunteer either touched the plate or sat with their legs pressed against the table creating a negative pressure on the balance; and on two occasions they ate in a rush). Finally, the investigator inadvertently set the balance to zero at the incorrect time on one occasion resulting in one additional invalid output. Taken together, the rate of non-usable UEM outputs amounted to 13% (19/149). Occasionally, participants unconsciously applied slight pressure on the table however this was not sufficiently strong to invalidate the UEM measures (it did not affect the weight recordings).

### Memory reconstruction test performance

**Fig. 6:**
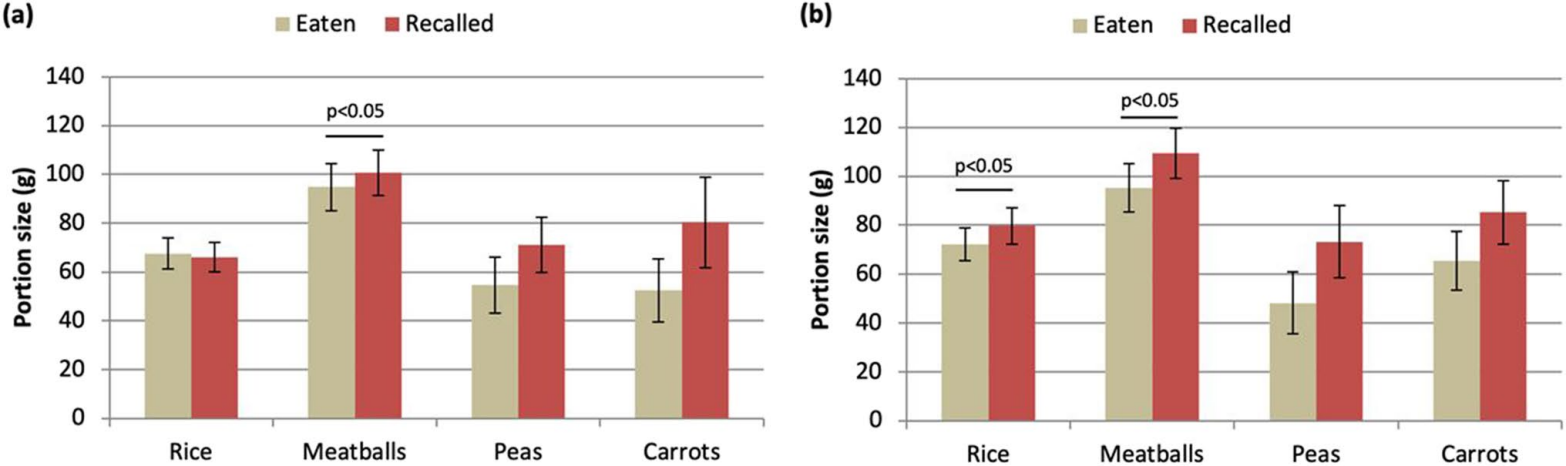
Results from the memory reconstruction task for the first 20 subjects completing the study. Bars depict the comparison of eaten vs. recalled portion sizes after using each plate. Data are means ± SEM. **a** Calibrated plate. **b** Control plate

A total of 148 outputs were recovered (76 from visit 1 and 72 from visit 2). There were four invalid outputs, two at each visit, due to protocol deviations (wrong interval time applied between the meal and the test); and four missing outputs in visit 2 due to participants failing to attend the session. The portion-size memory assessment showed 99% efficiency across the 76 participants (i.e., there was only one software failure across 148 collected outputs). No participants reported any difficulty in using or understanding the task. A preliminary (interim) analysis across the first 20 volunteers showed good memory recall for all foods but significant differences were detected between recalled and consumed portion sizes for meatballs for both plates and for rice for the control plate using unadjusted paired samples *t* tests/Wilcoxon tests (*p* < 0.05) (Fig. 6). These within-subjects differences disappeared in the final dataset including all 76 subjects, when analyzed using mixed effects linear regression (data not shown). No significant differences were detected between plate conditions for any foods in the initial (*n* = 20) or the final analyses (*n* = 76; *p* > 0.05 for all comparisons).

### Feasibility of blood extraction protocol

Overall, the integration of the blood extraction protocol with the other measurements proved feasible, however, it required meticulous time keeping and good coordination by team members as processing of blood samples for ghrelin analyses requires the addition of a protease inhibitor immediately after drawing each sample (therefore requiring at least two investigators at that point in time). Blood samples could be recovered from all 31 women for both visits, except for two participants who attended only the first visit.

#### Time required for blood extractions while participants were eating

Within the first 20 minutes of the meal, 100 extractions were taken from 31 volunteers (58 extractions at 5 min and 42 at 10 min). There were no extractions that coincided with eating beyond the 20 min. The mean ± SD time required for the nurse to perform the 5’ and the 10’ extractions together was of 167 ± 83 s (calibrated plate 144 ± 56 s, *n* = 29; control plate 188 ± 98 s; *n* = 31 participants). These periods represent on average 27% of the total mealtime (24% and 30% for the calibrated and control plate, respectively). To avoid erroneous calculations in UEM and gaze movement parameters, these periods of time were excluded from the respective meal micro-structure and gaze analyses.

#### Compliance with blood extraction target times

A total of 295 blood samples were drawn from the 31 participants across the two sessions, covering six extraction times: at 0 min (fasting), and then at 5, 10, 30, 60, and 90 min after finishing the meal. The mean (± SD) time difference between the target and actual extraction time was – 13.0 ± 54.4 s. This indicates that on average, blood draws were taken 13 s later than the stipulated time across the six extraction times although with some variability. By plate condition, the differences were of similar magnitude and not statistically significant (*p* = 0.67) (Table 1).

**Table 1:** Mean ± SD and 95% confidence interval (CI) for the differences between target and actual extraction times (in s), for extractions carried out in 31 volunteers at 5, 10, 30, 60, and 90 min after starting the meal

	Mean ± SD (s)	95% CI
Both plates (*n* = 295 measures)	– 13.0 ± 54.5	(– 6.8 to – 19.2)
Calibrated plate (*n* = 140 measures)	– 9.4 ± 46.8	(– 1.7 to – 17.2)
Control plate (*n* = 155 measures)	– 16.3 ± 55.2	(– 7.6 to – 24.9)

**Fig. 7:**
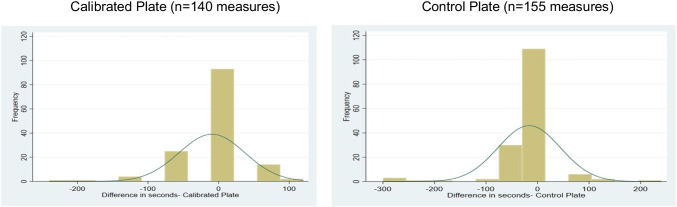
Frequency distribution of the differences between target and actual extraction times for extraction times at 5, 10, 30, 60, and 90 min for 76 volunteers. Values on the *X*-axis are seconds

Much of the deviation was driven by a few measures representing around 6% of the total samples. Thus, there were eight samples (2.7%) taken between 180 and 300 s later than the target time; and ten samples (3.4%) taken between 120 and 180 s later. The main reasons for time deviations were the obstruction and displacement of the intravenous cannula due to natural movement of the participant's arm during the meal. The remaining 277 samples (94%) were taken less than 2 min from the stipulated time, with 75% of the total (220 samples) taken less than 1 min away from the target (Fig. 7).

#### Impact of blood sampling on UEM measures

Table 2 shows the results of the mean values for each meal eating parameter between women providing blood samples and those not providing blood samples, after excluding extraction times in the first group. In agreement with the literature (Laessle et al., 2007), initial exploration of the data suggested a possible impact of BMI category on meal eating parameters such as bite size and meal duration. Results are therefore presented separately by BMI group. In the present study, the only parameter affected by blood draws was bite size, where the mean (± SEM) value in those not providing blood samples was slightly lower across both plate conditions than for those providing blood samples (with blood draws: 3.12 ± 0.19 g vs. without: 3.95 ± 0.17 g; *p* < 0.01). There was no impact of the blood draws on bite size in women with overweight or on any other parameter in any other women.

**Table 2:** Mean ± SEM for meal eating parameters collected with the Universal Eating Monitor (UEM) across women providing blood samples and those not providing blood samples, by BMI category. Data were pooled across both plate conditions to increase power

	Group with blood draws	Group without blood draws	*p* for difference
**Women with normal weight**	*n* = 26	*n* = 33	
Eating rate (g/min)	32.5 ± 1.8	32.3 ± 1.7	0.932
Bite size (g)	3.12 ± 0.19	3.95 ± 0.17	**0.002**
Deceleration rate (g/s^2^)	0.0011 ± 0.0001	0.0010 ± 0.0001	0.890
Meal duration (min)	11.0 ± 0.8	10.5 ± 0.6	0.633
**Women with overweight**	*n* = 29	*n* = 24	
Eating rate (g/min)	35.23 ± 2.62	32.60 ± 1.53	0.399
Bite size (g)	3.42 ± 0.16	3.47 ± 0.20	0.875
Deceleration rate (g/s^2^)	0.0012 ± 0.0002	0.0010 ± 0.0001	0.345
Meal duration (min)	9.73 ± 0.52	10.07 ± 0.48	0.634

### Eye tracking data analysis and data quality

A total of 149 video-recordings were collected with the Tobii eyetracker, 76 for the first visit and 73 for the second visit. Video-recordings of the second visit for three volunteers could not be collected due to these subjects failing to attend visit 2. In addition, for one volunteer, recordings for both visits were invalid because the participant mixed the food components on the plate before taking the first bite, effectively eliminating the AOIs.

Gaze capture across the original 149 recordings ranged from 24 to 96%, with a mean (±SD) of 83.1% (±11.8%). In total, 109 recordings (73%) provided a gaze capture of ≥ 80%. For reference, 134 recordings (90%) provided a gaze capture of ≥70%. In all, 58 volunteers (76%) produced recordings with gaze capture values of ≥ 80% across both visits.

#### Piloting and inter-rater reliability test for video analysis protocol (Lightworks 14.0)

Initial pilot testing of the manual protocol in Lightworks 14.0 across six representative recordings (three subjects consuming the same meal on both plate types), revealed the vegetables AOI as the one with the highest number of fixations, probably related to the larger surface of this AOI compared with the meatballs and the rice, especially for the calibrated plate. This tool limits the amount of carbohydrate and protein food to about ¼ of the plate for each macronutrient, while it guides on filling up half of the plate with vegetables (Figs. 2 and 4; and Supplementary Fig. S2).

Gaze capture of the six recordings ranged between 87 and 94% confirming good image quality. All volunteers followed the protocol correctly (did not mix the foods). Mixed zone fixation time across these six recordings accounted for < 8% of total fixation time with a mean (± SD) of 3.2 ± 2.5 % (range 1.3–7.5%)^.^ Values were of a similar magnitude across plate conditions (1.3–7.5 % for the calibrated plate and 1.3–3.1 % for the control plate). To put this into context, mean (± SD) % fixation times for the vegetables, rice and meatballs was 29.8 ± 7.8, 27.2 ± 8.0 and 14.0 ± 6.1%, respectively, in the same sample of videos. At this stage, the protocol included instructions for coding seven AOIs corresponding to vegetables, rice, meatballs, mixed food zones, plate border, empty plate and bread. Coding challenges identified included the mixed food zones, vegetables, border of the plate, sauce and dynamic gazes (e.g., volunteer looking at the food on the fork while the fork was being moved). Instructions were improved for the coding of these areas on both the main protocol and the AOI Visual Coding Guide. Following this step, the complete protocol and AOI visual coding guide were tested for inter-rater reliability (IRR).

**Fig. 8:**
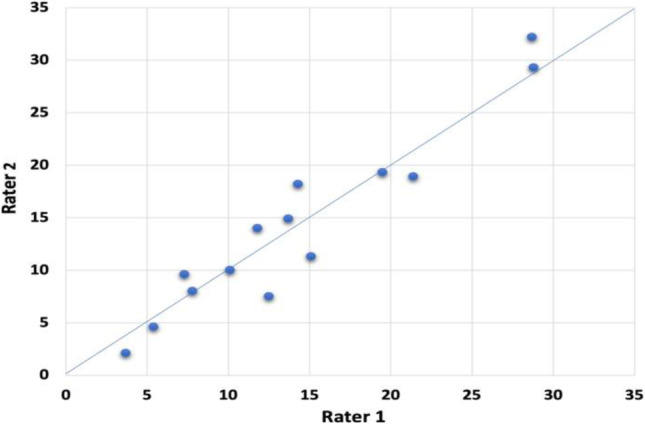
Correlation for 14 pairs of data between proportional fixation times (% time on each AOI relative to total AOI time) as registered by two independent raters using the manual coding protocol in Lightworks 14.0 across two video-recordings

For the IRR, two trained independent raters coded a total of 29,744 frames across two videos corresponding to the same volunteer eating with both plates. The analysis included 15,946 frames coded by rater 1 and 13,798 frames coded by rater 2. Therefore, 1189.76 s were analyzed (638 s for rater 1 and 552 s for rater 2). The video featuring the calibrated plate had about 9 min and the one with the control plate about 8 min of valid AOI data. Each rater coded the seven pre-established AOIs: rice, vegetables, meatballs, empty plate, border, bread and mixed zone, resulting in paired data for 14 AOIs (total *n* = 28). The largest differences in percentage (%) proportional fixation time were observed for the vegetables, rice and the mixed zone AOIs, while the smallest differences were observed for the border, empty plate and bread. There was very little variation between plate conditions (Table 3). The Pearson´s correlation coefficient was *r* = 0.96 (*p* < 0.001) indicating a strong correlation between raters (Fig. 8).

**Table 3:** Results of the reliable change index (RCI) calculations for paired data from two independent raters. A total of 14 AOIs were analyzed across two videos (A, with calibrated plate; B, with control plate). *Abbreviations*: SEm, standard error of the measurement; Sdiff, standard error of the difference between the two ratings. RCI, reliable change index. (*) Indicates significantly different ratings between raters (RCI > 1.96) with *p* < 0.05

AOI	Video Recording	Rater 1 (% fixation time)	Rater 2 (% fixation time)	SEm	Sdiff	RCI
Rice A	1	28.8	32.2	2.16	3.06	1.113
Meat A	1	7.3	14	2.16	3.06	2.193*
Veg A	1	14.3	14.9	2.16	3.06	0.196
Mixed A	1	15.1	10	2.16	3.06	– 1.669
Border A	1	5.4	2.1	2.16	3.06	– 1.08
Empty A	1	21.4	19.3	2.16	3.06	– 0.687
Bread A	1	7.8	7.5	2.16	3.06	– 0.098
Rice B	2	28.7	29.3	2.16	3.06	0.196
Meat B	2	11.8	9.6	2.16	3.06	– 0.72
Veg B	2	13.7	18.2	2.16	3.06	1.473
Mixed B	2	10.1	8	2.16	3.06	– 0.687
Border B	2	3.7	4.6	2.16	3.06	0.295
Empty B	2	19.5	18.9	2.16	3.06	– 0.196
Bread B	2	12.5	11.3	2.16	3.06	– 0.393

For the intra-class correlation (ICC), first, the percentage (%) proportional fixation time for each AOI for each rater was calculated (Supplementary information Table S1). From these data the ICC was computed at 0.97 (90% CI 0.94, 0.98), which can be interpreted as an excellent correlation (Koo & Li, 2016). Using this ICC value, the RCI were calculated for each paired AOI values**.** The SD *between subjects* used for calculating the RCI was 11.74 based on the sample of *n* = 14 pairs of data (see Supplementary information). Table 3 shows the results of the RCI and % proportional fixation time analyses for each AOI by rater. Values marked with an asterisk (*) are significantly different between raters (meatballs in the calibrated plate). Overall, the protocol was highly reproducible.

Although the mixed food zones were comparable between the raters, they still posed difficulty for coding when more than one food fell within the gaze point in different proportions, producing inconsistent ratings (visualized in the RCI for calibrated plate, − 1.70). For this reason, and because mixed zones accounted for a small proportion of the total time, this AOI was eventually re-coded as a single AOI for the validation analysis vs. AGM. To diminish differences in the meatball AOI zones (RCI for calibrated plate, 2.193) and vegetables (RCI for control plate, 1.47) the coding instructions for these AOI were improved in the final version of the protocol which was then used to develop the AGM protocol (see next section).

#### Piloting and validation of automatic gaze mapping (AGM) protocol (Tobii Pro Lab)

The first AGM protocol version was developed including the four selected AOIs using the manual protocol: rice, vegetables, meatballs and border of the plate. Because the AGM method treats any mixed food zones as the nearest AOI and any fork zones as the AOI that is behind the fork, it can create discrepancy vs. manual coding. To make the two methods compatible, we adjusted the Lightworks protocol so that mixed food zones and AOIs including the fork were recoded as the corresponding AOIs in the AGM method. By doing this we lost precision (i.e., some areas may be slightly over or underestimated if they are in a mixed food or fork AOI), however these AOIs accounted for < 10% of the total fixation time. Given the advantages offered by the AGM method, we considered this limitation as acceptable in the context of this study.

To account for changes in AOI size and shape (related to food disappearance), we integrated dynamic AOI coding in the AGM protocol and applied it to a sample of 10 representative videos previously coded in Lightworks by two trained raters. Table 4 shows the results of the validation analysis. Coding of vegetables and meatballs AOIs were similar across methods but the AGM method underestimated the rice AOI by about 4 s on average and the border AOI by about 3 s.

**Table 4:** Mean (± SEM) fixation times in seconds, obtained for AOI using the manual (Lightworks 14.0) vs. automatic gaze mapping (Tobii Pro Lab) coding protocols across a sample of ten video recordings (five subjects eating with an intervention plate), encompassing a total of 960 frames. Total number of frames in AGM also include non-coded frames in Lightworks

	Manual (total frames *n* = 352)		AGM (total frames *n* = 608)		*p* for difference
	Mean	SEM	Mean	SEM	
Rice	13.03	± 2.43	8.89	± 2.14	**0.032**
Meatballs	5.73	± 2.10	4.14	± 1.76	0.121
Vegetables	10.19	± 1.77	8.29	± 1.78	0.250
Plate border	1.52	± 0.37	4.01	± 0.91	**0.019**

**Fig. 9:**
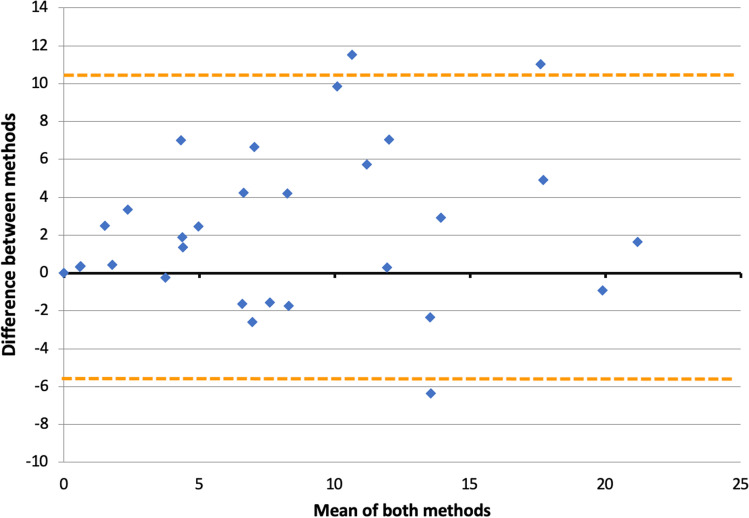
Bland–Altman plot of AOI fixation times (s) collected with two methods (manual coding vs. automated gaze mapping) over the first 60 min upon starting the meal. Differences between methods are plotted against the mean of both methods for the vegetables, rice, and meatballs AOIs across a sample of ten recordings (five subjects using both plates each; *n* = 30 pairs of data). *Dotted lines* indicate the upper and lower limits of agreement, respectively. The two values with a difference > 10 in the *Y*-axis correspond to the rice AOI for a single subject (both plates). The value < – 6 in the *Y*-axis corresponds to the vegetables AOI for another subject (calibrated plate). Both subjects were female, with overweight

Differences between methods were normally distributed therefore agreement between methods was explored using Bland–Altman plots for the rice, vegetables, and meatballs AOIs. This analysis showed that despite differences for the rice, the AGM protocol was largely comparable with the manual protocol taking into account that mixed food areas and AOIs including the fork may lack some precision when applying automatic coding (Fig. 9). This was confirmed by the ICC calculated across the 30 AOIs, which was high at 0.85 (90% CI 0.74, 0.92).

## Discussion

Research on human eating behavior has progressed significantly in the last 20 years thanks to the development and application of a range of approaches that are grounded in psychology and physiology (Allison & Baskin, 2009; Almiron-Roig et al., 2017; Yeomans, 2018). Nevertheless, because of the underlying complexity, an integrated approach is needed to gain a better understanding of the mechanisms involved in overeating and obesity. With this in mind, we have described a new methodological platform that provides this solution – an integrated/synchronized approach to real-time data collection, focusing on gaze movement, meal eating behavior, episodic memory, and satiety (both subjective and physiological).

Together, our observations demonstrate the feasibility of combining eye-tracking technology with meal micro-structural analyses (meal eating behavior), memory assessment and subjective and hormonal satiety responses to a portion-controlled meal. Subjects included women with overweight and obesity, plus lean men and women. While the results of the trial are beyond the scope of this paper (and will be published separately), preliminary findings suggest the calibrated plate had a positive effect on portion size control across all subjects, confirming the validity of the present methodology across different BMI categories and sex group (Vargas et al., 2019). Initial results also suggest that sensory (visual cues on the plate), cognitive (satiety expectations) and physiological mechanisms (changes in cephalic hormonal responses) may act together during the first 10 min after starting the meal, and result in particular meal eating behaviors (i.e., slower eating rate, smaller bite size, increased satiety), at least in some individuals. The nature and temporal association of these processes could not have been determined using measures captured in isolation, or with exclusively subjective measures, which highlights the unique benefits of this approach.

To our knowledge, this is the first methodological platform specifically designed to simultaneously combine these measures during actual food consumption. While physiological measures have previously been used in combination with UEM recordings (Näslund et al., 1998), studies examining gaze movement during a real food intake test are scarce (Gough et al., 2018) or have used less precise methods and different populations, e.g., filming in babies (McNally et al., 2019) . A recent study in adults (Wang et al., 2018) explored visual attention paid to food during self-serving in a real-life buffet context, however, gaze movements were not measured during actual intake and physiological parameters were not monitored. None of these studies measured gaze movements in combination with the UEM though.

The lack of related research probably reflects the inherent difficulty in collecting multiple behavioral and physiological variables in real time, many of which have the potential to interact and be subject to bias due to study demand (Best et al., 2018; Yeomans, 2018). In addition, analyzing video data from wearable devices is complicated because gaze position is recorded in reference to the users point of view, rather than to the target stimulus (MacInnes et al., 2018). This means that natural head movements during eating will interfere with feature matching algorithms in AGM. A further challenge is the collection of gaze data in response to dynamic stimuli changing in size and shape (such as food disappearing from a plate), something not encountered with static food images or constant-size stimuli shifting position (Holmqvist & Andersson, 2017; van der Laan et al., 2017; Werthmann et al., 2011).

### Strengths and limitations of this study

To address and minimize the impact of the challenges inherent to the realization of this work, we adopted a series of precautions. First, we employed a reasonably large sample size, including men and women between 18 and 60 years, with varying body weights, to be able to establish method validity across a range of subjects. Due to the outbreak of the COVID-19 pandemic, the full sample of men could not be recruited. This limited power for some sub-group analyses (e.g., comparison of bite size by sex group required a minimum of 23 men); however, we observed significant differences in portion size selection in the men sample and the validity and reliability analyses remained sufficiently powered as all subjects were considered together.

To diminish hypothesis awareness, participants were told the study was about healthy eating. Despite not mentioning portion size, this message may have prompted differences in portions selected as some may be knowledgeable about the importance of portion size in relation to healthy eating as well as potential for selection bias including weight- or health-conscious participants. Our data on portion size norms suggest that participation effects were present (i.e., subjects reported self-serving less amount in the lab than habitually at home), which needs to be considered when interpreting the results.

Second, we applied a combination of analyses to establish inter-rater reliability and method agreement, and selected representative individuals from the larger sample when analyses of complete datasets were not feasible within a reasonable timeframe (e.g., manual coding of eye-tracking video-recordings). As part of a sensitivity analysis, we included a sub-sample of volunteers not providing blood samples, which allowed us to isolate any potential effects of the blood sampling procedure on meal eating parameters. Third, we considered the impact of protocol deviations and quantified their effect. For example, we explored deviations from target times for blood extractions (*n* > 200 measures) based on time-dependent outcomes. Finally, we obtained measures for the performance of the various devices and software and compared these with previously published data. Overall, our methodological platform performed well and, in some cases, with less error rate than previous versions.

While a limitation of the present platform is that some of its components are inevitably limited to laboratory settings (e.g., UEM), where people may know they are being closely observed and may change their behavior, other components have the potential to be used in free-living studies and allow more naturalistic measurements (e.g., eye-tracking glasses). The Tobii Pro Glasses 2 belong to the latest generation of portable eye-trackers, which allow free head movement while interacting with real-world stimuli. They have a reported accuracy and precision close to other wearable eye-trackers (MacInnes et al., 2018), and performed well in our study, resulting in more than 70% of recordings with a gaze capture of 80% or higher (see below under *Validity of eye-tracking measures)*. The Tobii Pro Lab AGM software integrates feature matching algorithms that allow dynamic AOI analysis based on the matching of salient features of the target stimulus in a reference image and image data recorded with the eye-tracker world camera (front-viewing). The combination of these two components thus represented a good match with the current study design, featuring consumption of real food. Despite this, a number of limitations needed attention. In particular, AOI-based analyses using hand-drawn AOIs can easily lead to false-positives in stimuli containing closely located AOIs such as the menu used in this study (Orquin et al., 2016). This technique relies on subjective location, size, and shape determination of the AOIs (Hessels et al., 2016). If hand-drawn AOIs are used for automated coding, the coding process requires the generation of several template images depending on the distribution of foods on the plate (dynamic AOI analysis), which complicates the process.

The optimized UEM resulted in a much lower proportion of erroneous outputs compared with the traditional version, where the balance is directly attached to the surface of the table, therefore allowing vibrations from the participant to directly transfer to the balance (Kissileff et al., 1980). Our previous study using the traditional UEM reported an error rate of 33% due to a combination of scale reading faults and participants unconsciously unsettling the equipment (Almiron-Roig et al., 2015). This is more than double the error rate observed in the present study with the optimized UEM (13% error rate including 8% error due to scale reading faults). Another advantage of our current platform is that it allows covert observation of participant´s actual behavior on the UEM thanks to the eye-tracker world camera. This allows us to identify issues related to the UEM, and also to other study procedures in real time, helping to optimize study protocols. Despite these advantages, the optimized UEM remains limited in its portability and size, and users are required to sit and eat in a particular way. More advanced technological solutions that are smaller, more mobile, flexible and concealable are therefore needed to widen the range of applications.

Inclusion of blood extractions synchronized with eating behavior measures posed a challenge when extraction times coincided with the time recordings for the UEM and the eye-tracker. It is likely that introducing pauses during mealtimes and the actual extraction process affects natural eating behavior and so considering this interaction is important. While laboratory conditions are traditionally considered low naturalistic environments, potentially affecting food intake measures (Robinson et al., 2015; Robinson, et al., 2014b), highly controlled conditions are still necessary in order to distinguish the effects of specific interventions from other factors (Hetherington & Rolls, 2018; Yeomans, 2018).

Blood extractions had a significant impact on only one UEM parameter (bite size), and only in non-overweight women. However, the impact was small, at less than 1 g. For reference, average bite size in this study was in the range 3.5 to 4.0 g per bite across both plate conditions. Despite this, it seems relevant to consider participant characteristics such as BMI, when combining blood drawings and UEM measures and to adjust timings to avoid draws that coincide with eating whenever possible. This will also result in a more naturalistic eating experience for the volunteer.

Total blood extraction time was similar in both plate conditions, suggesting that the extraction procedure per se does not condition or elongate meal duration differentially by testing conditions. This was supported by the high proportion of valid UEM outputs amongst participants providing blood samples (54/60 outputs, or 90%). In about 25% of the samples we detected some deviation from target extraction times above the recommended 30-s interval used in a previous study involving similar measures (Yeomans et al., 2016) although 75% of the samples were taken < 1 min away from the target time and 94% < 2 min away. Overall, these results suggest blood extractions are compatible with the other protocol components however tight attention to extraction times is necessary, especially if short-acting biomarkers are being measured which may peak within the first 10 min after starting the meal (Smeets et al., 2010).

Finally, results from the memory reconstruction tool showed acceptable correspondence between portions selected using this software and portions consumed ~3 h earlier. On this basis we can be reasonably confident that the tool provides a way to record memories for portion size, although inter-subject variability was high. Moreover, because this correspondence was preserved across food types and serving plates (in the final sample), it would seem likely that the software can be applied in a variety of contexts. In part, this success is likely to be because the process of creating a portion on a screen is designed to simulate the process of real-world portion-size selection which, itself, is a highly practiced daily behavior. Consistent with this idea, most participants reported that they felt the task was intuitive and easy to complete.

### Validity of eye-tracking measures of actual food intake

It is not uncommon for eye-tracking studies to suffer from issues threatening their internal and external validity. Examples of such threats include appropriateness of the comparison; multiple metric analysis; data quality; software and other defaults (e.g., filters and cut-offs); fixed vs. free exposure times; data interpretation and data extrapolation (external validity) (Orquin & Holmqvist, 2018).

Appropriate comparisons include stimuli which are expected to elicit similar responses across different types of subjects as opposed to stimuli greatly differing in complexity or familiarity as these would involve different causes for attracting attention. The present study includes two very similar stimuli (two plates of the same size and material, on which the same three or four foods are placed each time, adjacent to each other), differing only by the quantities of each meal component and their location on the plate (see Supplementary Fig. S2). We expect these stimuli to be able to elicit sufficiently similar responses across subjects of different characteristics and therefore allow for a meaningful comparison. In addition, we included sufficient sample size to explore how subjects of different gender and body weight may respond to each of the plates independently.

Orquin and Holmqvist (2018) caution about the use of multiple metrics as these tend to be highly correlated and advise to work with pre-formulated hypotheses to guide on the specific metric of interest. Total dwell times also need to be used cautiously to avoid inappropriate aggregation of data (e.g., specific AOIs may receive more or longer fixations because of difficulty or complexity instead of salience). To account for differences in meal duration across subjects, the present study used proportional dwell time for each AOI over the course of the meal as the main metric. This metric was used to calculate the sample size as part of the main study hypothesis.

Data quality is highly related to the precision (reliability), avoiding false-positives (accuracy) and sensitivity of the eye-tracker. So far, no predetermined standards for sensitivity for a given eye-tracker´s accuracy and precision exist but *capture rate* (the percentage of fixations that fall within the boundaries of that object) is commonly used as a quality measure (Orquin & Holmqvist, 2018). Ideal gaze capture thresholds for eye-tracking data typically depend on the context and purpose of the study (Holmqvist & Andersson, 2017). Assuming that saccades may constitute between 5 and 15% of all eye movements in activities such as reading, about 85–95% of gaze samples could be categorized as belonging to fixations (Hvelplund, 2014). Taking into account our within-subjects experimental design, a cut-off value of 80% in gaze capture seems justifiable. Based on this threshold, the majority of our video-recordings could be considered of sufficient quality, however, very low gaze capture in a few recordings imply that some may need to be excluded. Low gaze capture may result from incorrect calibration vs. actual distance to the stimuli (MacInnes et al., 2018), incorrect positioning of the eye-tracking glasses (i.e., resulting in the front-view camera being directed away from the AOIs) or inherent inaccuracy (e.g. participants with deficient visual acuity, uncontrolled head movement) (Thibeault et al., 2019). We screened participants for sufficient visual acuity and performed individual calibration for each subject in the actual testing room, following the manufacturer’s instructions which involve a one-point calibration method with a card placed at comparable distance to the actual stimuli. Other devices use a more extensive 3 to 9-point calibration method and are reported to have higher accuracy and precision (MacInnes et al., 2018). In our study, 73% of recordings provided 80% or more of gaze capture. Based on the sample size (*n* = 149 recordings) we believe this limitation is acceptable, however, pre-test point-to-point and trial-to-trial variability checks would probably improve accuracy and are recommended (Thibeault et al., 2019).

Using a high cut-off for minimal fixation durations may impact data validity if it results in loss of fixation data. For example, using a 200-ms cut-off may result in around 50% of fixations being lost if we consider that the median of a fixation duration distribution lies between 200 and 300 ms (Orquin & Holmqvist, 2018). We applied a cut-off of 100 ms (Duchowski, 2017; Werthmann et al., 2011), because saccades rarely exceed this duration (Fuchs, 1971).

The current study design employed free exposure time. Actual exposure time depended on how long the subject took to consume the food on the plate and therefore differed across participants. Free exposure times avoid effects of time pressure or idleness (during which the subject may stare at any object at random or continue in a post-decision state) (Clement, 2007). Therefore, free exposure times are recommended but adjustments are required to make data comparable across subjects (Orquin & Holmqvist, 2018). We adjusted dwell times to each subject´s meal duration and limited the analysis to the first 60 s after stimulus onset. This is longer than those used previously (e.g., 0.5 s) (Shimojo et al., 2003), reflecting differences in main study metrics and overall study purpose. In our case, the average meal duration for participants was 10 min so we chose what we considered a sufficiently meaningful interval to analyze.

Finally, data interpretation needs to take into account the appropriateness of data aggregation, especially if total dwell time is used to draw conclusions about which AOI receives more attention. For example, differences in fixation duration may respond to goal-driven (i.e., relevance) as opposed to stimuli-driven (i.e., salience) fixations (Orquin & Holmqvist, 2018). In the present study, participants were not asked to complete any cognitive task after viewing the meal or while eating it. Initial processing of the food and plate stimuli likely took place before eating as each participant pre-selected their own foods from an identical buffet across both sessions.

We therefore consider that dwell time will mostly reflect salience of the foods placed on the plate, or of the plate itself. Data obtained with the AGM method allows for detailed analyses, which can be performed in the future to investigate salience vs. relevance effects. Importantly, fixation time is a mere surrogate of attention or salience, as eye-trackers only collect eye movements and gaze data. While eye movements are closely coupled with attention they are not perfectly aligned (Deubel, 2008). During an eating event there may be idle times when the subject may not be fixating specifically on any food (Wansink, 2010), yet the eye-tracker will detect a fixation on that AOI. This will lead to fixated but not processed stimuli (false-positives). Peripheral processing on the other hand (that is, identifying an object without being conscious of it) may lead to false-negatives (stimuli processed but not fixated).

To decrease false-negatives, we employed a highly controlled eating environment devoid of distractions and where all relevant stimuli were confined within the plate area. We also strived to define AOIs as accurately as possible, using a narrow margin given the close proximity of AOIs (Orquin et al., 2016), and repositioned AOIs at regular intervals in the AGM method to avoid false-positives. The AGM method proved comparable to our gold standard method (manual coding), suggesting that the current settings in the Tobii ProLab protocol are valid.

There are further limitations of using AOI-based algorithms for automatic gaze mapping (Salvucci & Goldberg, 2000). For example, such algorithms may label points outside the target areas as saccades instead of fixations, and thus eliminate relevant data. Fixation groups may also become removed if they are below a specific duration. Saccades present throughout large target areas may be identified as fixations, especially if data on time averaging is included. Despite these limitations, AOI-based dwell-time algorithms are valuable to help explain collections of fixations organized around visual targets and are good for aggregate analyses (Salvucci & Goldberg, 2000).

Overall, manual coding will overcome the problem of aggregate analyses, which may conceal informative low-level behavior not captured by AGM. However, manual coding is subject to error in human scanning behavior, which can affect consistency, accuracy and take a large amount of time. For this reason, automated eye movement analysis is recommended, however, due to the limitations noted above, model predictors need to be compared against individual trial protocols (Salvucci & Anderson, 1998).

In summary, the eye-tracking component of this study involved a number of challenges not typically encountered in traditional static scene viewing experiments. Perhaps the most relevant was that participants” head movements were not restricted to allow for natural behavior while eating. This resulted in frequent shifting of the AOIs, which required correction during AGM. Also important, the stimulus was highly dynamic as the amount of food on the plate gradually reduced over the course of the meal. Participants were instructed to avoid mixing the meal components in an attempt to allow for pooled AOI-based analysis and plates were placed in the same orientation as much as possible. Despite this, the coding necessitated frequent AOI adjustments due to the combination of head movements and the AOIs changing in size and shape over time. To improve accuracy, future tests should include a pre-test point-to-point and trial-to-trial variability analysis (Thibeault et al., 2019). Categorizing subjects according to natural head movement pattern while eating may also assist in feature matching AGM analysis.

## Conclusions

The present analysis of a novel methodological platform indicates that combining eye-tracking, an optimized UEM, and a memory test, alongside physiological measures of satiety, is feasible and it provides a detailed analysis of eating behavior processes in a controlled laboratory environment. Beyond optimization, its application requires validation in the target population and quality control of each component in the platform. Staff training is needed, especially to ensure low inter-rater error rates if more than one video data coder is involved. Furthermore, it is important to introduce measures to reduce variability in participant’s responses due to unfamiliarity with the equipment (e.g., by training participants), to physiological conditions, food preferences and/or the eating environment itself. Future applications of this platform should consider minimizing clashes between meal micro-structure and blood extraction timings and should apply pre-tests to estimate eye-tracker accuracy within the actual testing environment. Beyond food-related stimuli, the principles of this methodological platform are transferable to other contexts examining integrated real-time measures of human behavior.

## Supplementary Information

ESM 1 (DOCX 5112 kb)
