# Supplementary material for: Development and validation of a new methodological platform to measure behavioral, cognitive, and physiological responses to food interventions in real time
Source: Behav Res Methods. 2022 Jan 31;54(6):2777–801. doi: 10.3758/s13428-021-01745-9 (PMC8802991; doi:10.3758/s13428-021-01745-9)
Supplement: Supplementary file 1 — (DOCX 5112 kb) [file 13428_2021_1745_MOESM1_ESM.docx]

**SUPPLEMENTARY INFORMATION**

**Development and validation of a new methodological platform to measure behavioural, cognitive and physiological responses to food interventions in real time**

M.A. Vargas-Alvarez, H. Al-Sehaim, J. M. Brunstrom, G. Castelnuovo, S. Navas-Carretero, J.A. Martínez, E. Almiron-Roig

1. **Questionnaires**

**Habitual portion size / portion size norms questionnaire**

Four item 100 mm VAS (with vertical anchors at the extremes) questionnaire. Each question was adapted from a published VAS in Robinson et al. 2016^1^, which reads: “A normal serving of this meal would be (anchors: a lot smaller, a lot bigger)”.

For the purpose of this study, the above wording was adapted to:

“Compared with what I self-served today, a normal serving of this meal for me would be (a lot smaller, a lot bigger)” (Q1).

For Q2-Q4 “this meal” (*esta comida* in Spanish) was changed for “rice”, “vegetables”, and “meatballs” (*arroz*, *verdura* and *albóndigas*, respectively).

Spanish translation of the final questions:

*Q1. Comparado con lo que me he servido hoy, una ración normal de esta comida para mí sería (Mucho más pequeña-Mucho más grande)*

*Q2. Comparado con lo que me he servido hoy, una ración normal de arroz para mí sería (Mucho más pequeña-Mucho más grande)*

*Q3. Comparado con lo que me he servido hoy, una ración normal de verdura para mí sería (Mucho más pequeña-Mucho más grande)*

*Q4. Comparado con lo que me he servido hoy, una ración normal de albóndigas para mí sería (Mucho más pequeña-Mucho más grande)*

**Liking and Expected satiety for the meal**

Single 100 mm VAS (with vertical anchors at the extremes) adapted from Forde et al. (2015)^2^ and reading:

How filling do you think this meal is?” (Not at all-Extremely)

Spanish translation: *¿Cree que la comida que se ha servido es saciante? ¿Cuánto? (Nada-Mucho)*

How pleasant was this meal? (Not at all- Extremely)

Spanish translation: *¿Cuánto le ha gustado esta comida? (Nada-Mucho)*

**Subjective appetite**

Four item 100 mm VAS (with vertical anchors at the extremes) questionnaire based on the original VAS questionnaire developed by Hill & Blundell and validated in Flint et al. (2000) ^3,4^:

| English version | Spanish translation (adapted from SWEET Project, 2019)^5^ |
| --- | --- |
| Q1. How hungry do you feel? (Not at all-Extremely) | *¿Se siente usted hambriento? ¿Cuánto? (En absoluto-Totalmente)* |
| Q2. How full do you feel?  (Not at all-Extremely) | *¿Se siente usted lleno? ¿Cuánto? (En absoluto-Totalmente)* |
| Q3. How thirsty do you feel?  (Not at all-Extremely) | *¿Se siente usted sediento? ¿Cuánto? (En absoluto-Totalmente)* |
| Q4. How nauseous do you feel? (Not at all-Extremely) | *¿Siente usted náusea? ¿Cuánta? (Nada-Mucha)* |

**Portion tool acceptance**

Seven item 5-point Likert Scale questionnaire adapted from Almiron-Roig et al., (2016)^6^ where 1=Disagree completely (*Totalmente en desacuerdo*) and 5=Agree completely (*Totalmente de acuerdo*):

| English version | Spanish Translation |
| --- | --- |
| Q1. I like the presentation | *Me gusta el aspecto* |
| Q2. It would fit my lifestyle | *Encajaría con mi estilo de vida* |
| Q3. This tool fits with my family/home life | *Encajaría con mi vida familiar/doméstica* |
| Q4. I would feel embarrassed using this tool at home/ with others | *Me daría vergüenza usarlo en casa o con otros* |
| Q5. It seems easy to use | *Parece fácil de usar* |
| Q6. Looks resistant | *Parece resistente* |
| Q7. Seems practical | *Parece práctico* |

**Portion control self-efficacy**

Eight item 5-pt Likert scale questionnaire based on the validated portion control self-efficacy scale (PCSE) scale by Fast et al. (2015)^7^. The full scale score ranges from 1 to 40 points.

| English version*  (1=Strongly disagree; 5=Strongly agree) | Spanish translation  (1=Totalmente en desacuerdo - 5=Totalmente de acuerdo) |
| --- | --- |
| Q1. *I believe I can eat standard food portions when served portions that are too large* | *Creo que puedo comer porciones de comida de tamaño estándar cuando me sirven porciones demasiado grandes* |
| Q2. *I can handle eating the right food portions no matter what comes my way* | *Puedo encargarme de comer las porciones de comida adecuadas ante lo que surja.* |
| *Q3. I feel confident that I can leave food on my plate if I think a serving size is too large* | *Estoy segura/o de que puedo dejar comida en el plato si creo que el tamaño servido es demasiado grande.* |
| *Q4. When eating with others, they influence how much I eat* | *Cuando como con otros, éstos influyen en cuánto como.* |
| *Q5. It would be easy for me to control the size of the portions that I eat at social events or at home* | *Me sería fácil controlar el tamaño de las porciones que como en eventos sociales o en casa* |
| *Q6. I don't know if I can control the size of the portions that I eat at social events or at home.* | *No sé si puedo controlar el tamaño de las porciones que como en eventos sociales o en casa* |
| *Q7. I am confident that I can control the size of the portions that I eat when out with friends or when I eat at home with others* | *Estoy segura/o de que puedo controlar el tamaño de las porciones que como cuando salgo con amigos o cuando como en casa con otros.* |
| *Q8. I am confident I can judge whether a restaurant serving is appropriate when out* | *Estoy segura/o de que puedo juzgar si una porción de restaurant es apropiada cuando salgo con otros* |

*For the purpose of this study, the original English version was very slightly modified to refer to the home environment rather than the out-of-home environment (questions Q4-7, red text). These changes alongside the back-translated version were verified with the authors (J. Harman, personal communication).

1. **Protocol development stages for Lightworks 14.0 (manual coding)**

*Step 1: Definition of Areas of Interest (AOIs)*

Our study involved two contrasted composite stimuli located in the centre of the visual field and tested across a large sample of volunteers. We used Salvucci and Goldberg’s recommendations for AOI-based algorithms ^8^ and adapted them to manual coding giving emphasis to the temporal components (i.e. minimal fixation duration and local adaptability, that is, allowing to compensate for differences between “steady-eyed” subjects and those with large and frequent eye movements). Non-AOI based algorithms can identify fixations at any location in the visual field. In contrast this study sought to identify fixations that occur within specified target areas for which an AOI-based approach was a better fit. This method also allows transforming each fixation into a group (formed by collapsing consecutive fixation points for the same target) into a single record, thereby providing a more complete result than individual fixation analysis ^8^.

To account for potential limitations of AOI-based analyses, we defined the AOIs in Lightworks as close as possible to the actual margin of each food component that is, applying 0º of visual angle margins to avoid false positives ^9^.

To further optimise the application of the AOI method, we focused on the foods that were physically on the plate and excluded any foods that were outside the plate (i.e. condiments and bread). This resulted in only 3 meal components being analysed within each recording (1 for vegetables, 1 for rice and 1 for meatballs), in addition to the space occupied by the border of the plate, and the empty plate space that became visible as the volunteer started to consume the food. These five objects represented the first main AOIs coded (vegetables, rice, meatballs, border, empty plate). Any gazes outside these five AOIs were eventually disregarded. In addition, a proportion of gazes fell on the fork, with or without food on it, and another proportion fell on what could be called “mixed zones” between 2 AOIs (**Figure 5** in main manuscript). Both the fork and mixed zones could contain either two foods belonging to different AOIs, or one or more foods and the plate border, the empty fork or a non-coded area e.g. bread, condiments. The Tobii ProGlasses 2 display the gaze point as a circle superposed to the video images (**Figure S1b**), easily resulting in ambiguous coding for fork and mixed food fixations. An initial exploration of the magnitude of fixation time on mixed food and fork zones across 6 representative videos, indicated that neither of these zones accounted on average for more than 5% of the overall fixation time for the meal (mean ± SD mixed zones: 3.2 ± 2.5%; fork: 3.8 ± 2.4%). Considering this and that gazes on these particular AOIs were not a primary outcome of the study, the final protocol excluded the fork and mixed zone AOIs when these were impossible to assign to one of the main AOIs.

*Step 2: Selection of coding method*

Data coding in Lightworks can be performed by the *time stamp* method or the *frame counting* method. In the time stamp method, both the start and end points are registered for each frame in a 6 digit format (i.e. MM:SS.ff for minutes, seconds and frames, respectively). Videos created with 25 fps contain frames number 0 to number 24. In contrast, the frame counting method involves coding fixation times by counting and recording the number of frames during which the gaze point is inside each of the main AOIs. For this study, the frame counting method was selected as it was found to be less time consuming, and reproducible across independent assessors^[[1]](#footnote-1)^.

Quality control steps were applied to ensure there were no errors (see Step 6).

*Step 3: Setting up a threshold for minimal fixation duration*

This step is essential to remove raw saccade data points and collapsing raw fixation points ^8^, eliminating irrelevant information which typically occurs during saccades ^10^ and discarding smaller eye movements that occur during fixations, (e.g. tremors, drifts, flicks) ^11^. For videos at 25 fps, less than 3 frames (100 ms) are not considered meaningful ^12,13^, given that fixations tend to last between 200-400 ms while saccades rarely exceed 100 ms ^10^. Therefore, the minimal fixation duration was set at 100 ms or 3 frames.

*Step 4: Defining start and end times*

The start and end times were defined using the time stamp provided by Lightworks. The Start time stamp was defined as the time corresponding to the first frame when the volunteer was looking at the food on the plate (usually before picking up the fork). The Stop time stamp was defined as the time when the volunteer pressed the bell to call the investigator (first touch of the button). The first fixations were those made up by more than 2 frames (that is, 3 or more) after the Start time.

*Step 5: Assigning codes to each AOI*

To assign codes to each of the pre-defined AOIs we developed an AOI Visual Coding Guide (see **Section 3 below**), based on the proportion of each food included in the circular marker for the Tobii ProGlasses gaze point. A gaze point had to include at least 51% of a given AOI to be considered that AOI or when that was unclear, we considered the AOI corresponding to the centre of the circle. In addition, detailed instructions on how to code each of the AOIs were included in the protocol (e.g. the sauce was coded as part of the meatballs or the other foods if it had spread onto them).

*Step 6: Quality control*

Quality control measures were included in the protocol as the frame counting method can be prone to errors for large numbers of frames. Fixations longer than 25 frames were coded in duplicate using the time stamp method or double-counted to verify accuracy. In addition, for long fixation times of unclear target areas, the start frame was registered and that section of the video was examined at regular speed to get an idea of what the subject was looking at or intending to do.

*Steps 7 & 8: Data compilation and final checks*

Data on frame counts were compiled in an Excel template from which the total duration of the coded time was calculated and used to work out the percentage fixation times by AOIs. To obtain the duration in seconds after all frames were recorded and added, the number of frames was divided by 25. Following this, each Excel file was verified for completeness, noise data, errors and missing data. The main outcome explored from these data will be overall gaze dwell time for the meal and per AOI (how long looking at meal/AOIs). Further analyses may include additional metrics ^13,14^.

1. **AOI visual coding guide for Lightworks 14.0.**

The red circle represents the gaze point as captured by the Tobii ProGlasses 2. The cross ( ) represents the centre of the circle and thus the specific fixation point.

Orange = Food 1 (e.g. rice); (F1) Green pattern = Food 2 (e.g. veggies); (F2)

White = Empty plate/Sauce (E) Blue = Border (B)

1. **Inter-rater reliability tests for Lightworks manual protocol**

An initial inter-rater reliability (IRR) evaluation was conducted during spring 2019 on version 3 of the protocol. The kappa statistic from Viera & Garrett^15^ usually employed for inter-rater reliability assessments, could not be applied here because the data are not categorical (scores). Therefore, an alternative inter-rater reliability set of tests for continuous data was applied, based on the literature^16^. These tests include correlation, the intra-class correlation coefficient (ICC), and an agreement test (initially the Bland & Altman plot). The literature recommends using a set of tests, rather than a single one as correlation analyses on their own for example, are susceptible to systematic bias, while agreement and reliability do not always co-exist^17^.

For the intra-class correlation (ICC), first, the percentage (%) proportional fixation time for each AOI for each rater was calculated as shown in **Table S1**. The mean of each AOI rating between raters 1 and 2 for both the calibrated and control plate is represented as R1+R2/2. The B^2^ and W^2^ values represent the sum of the (Rating – Mean) values squared for the *Between* and *Within* calculations, respectively. The SD_b_ and SD_w_ values were verified in STATA v12.0 using a one-way ANOVA test producing similar resuts (SD_b_ of 11.63 and a SD_w_ 1.87).

From these data the ICC calculated at a 90% confidence level was 0.97 (CI 0.94, 0.98). The ICC was subsequently used to calculate the RCI, based on the procedures described in Stolarova et al. (2014)^16^. The sequence of calculations was performed in Excel and is included below.

For references for ICC see footnote^[[2]](#footnote-2)^

MAVA and HA stand for the initials of each rater.

1. **Supplementary Figures**

**Figure S1**. Study setting. (a) Example of tray featuring the study foods. Subjects had to select rice, meat and at least one of the vegetables. The bread, fruit and condiments were optional. Complimentary water was offered after the meal. (b) Example of scene from the eye-tracker front-viewing camera during an eating session.

| (a)  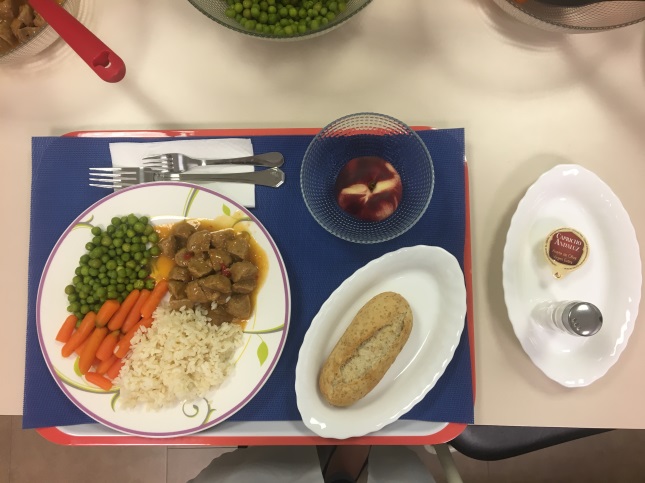 | (b)  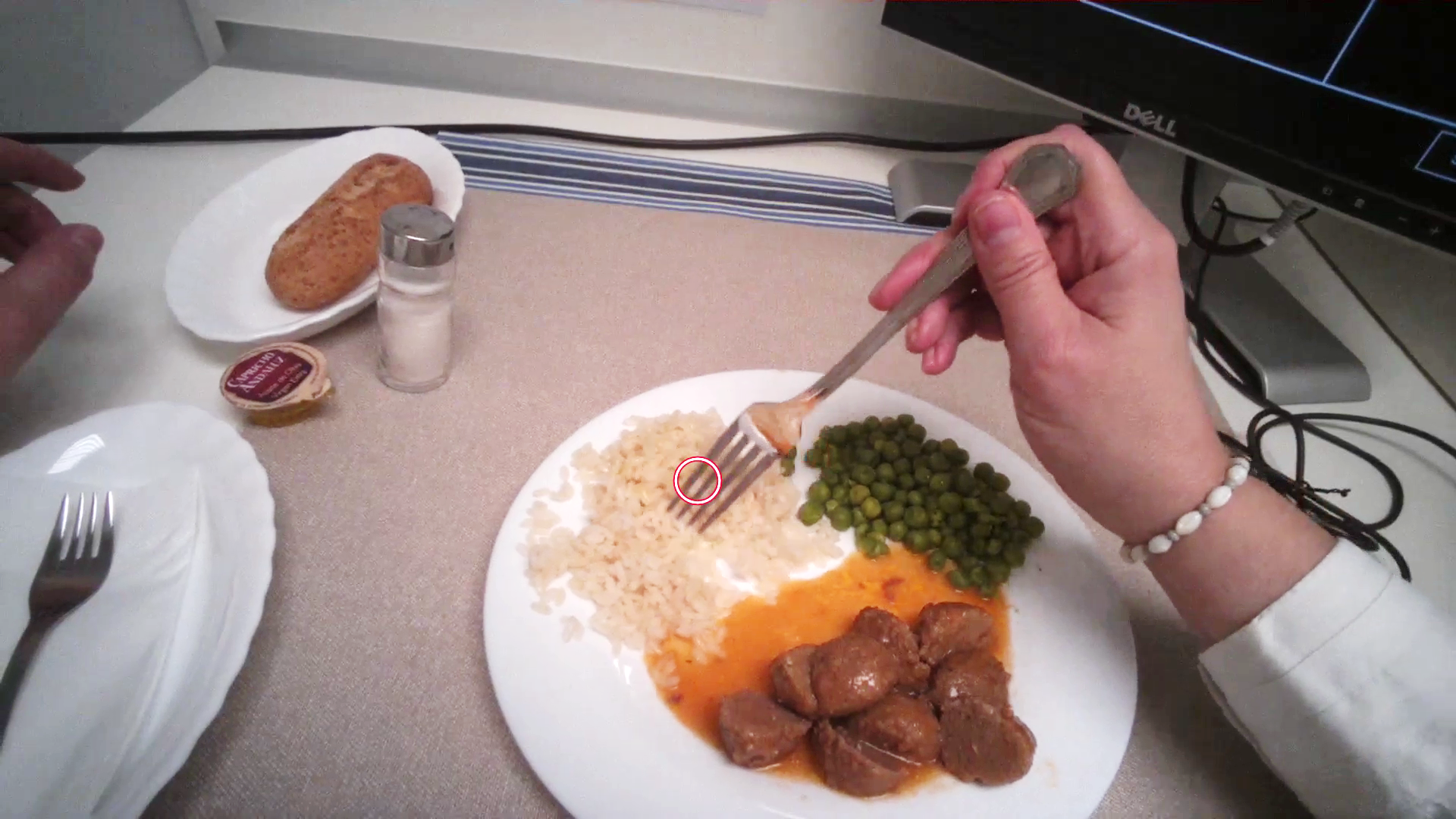 |
| --- | --- |

**Figure S2.** Examples of the plate stimuli used for analysis of gaze movements. (a) Meal served on calibrated plate. (b) Meal served on control plate.

| (a)  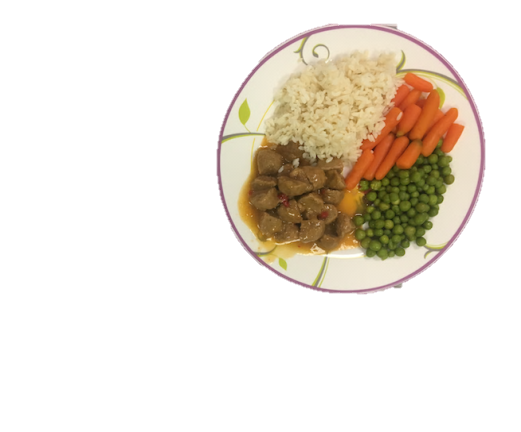 | 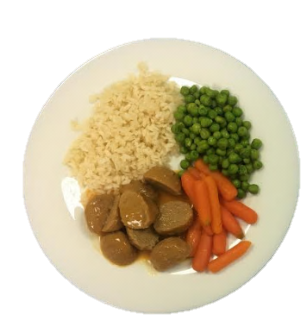(b) |
| --- | --- |

**Figure S3.** Screenshots of the memory software showing the memory task screens. English translations are available from the authors.


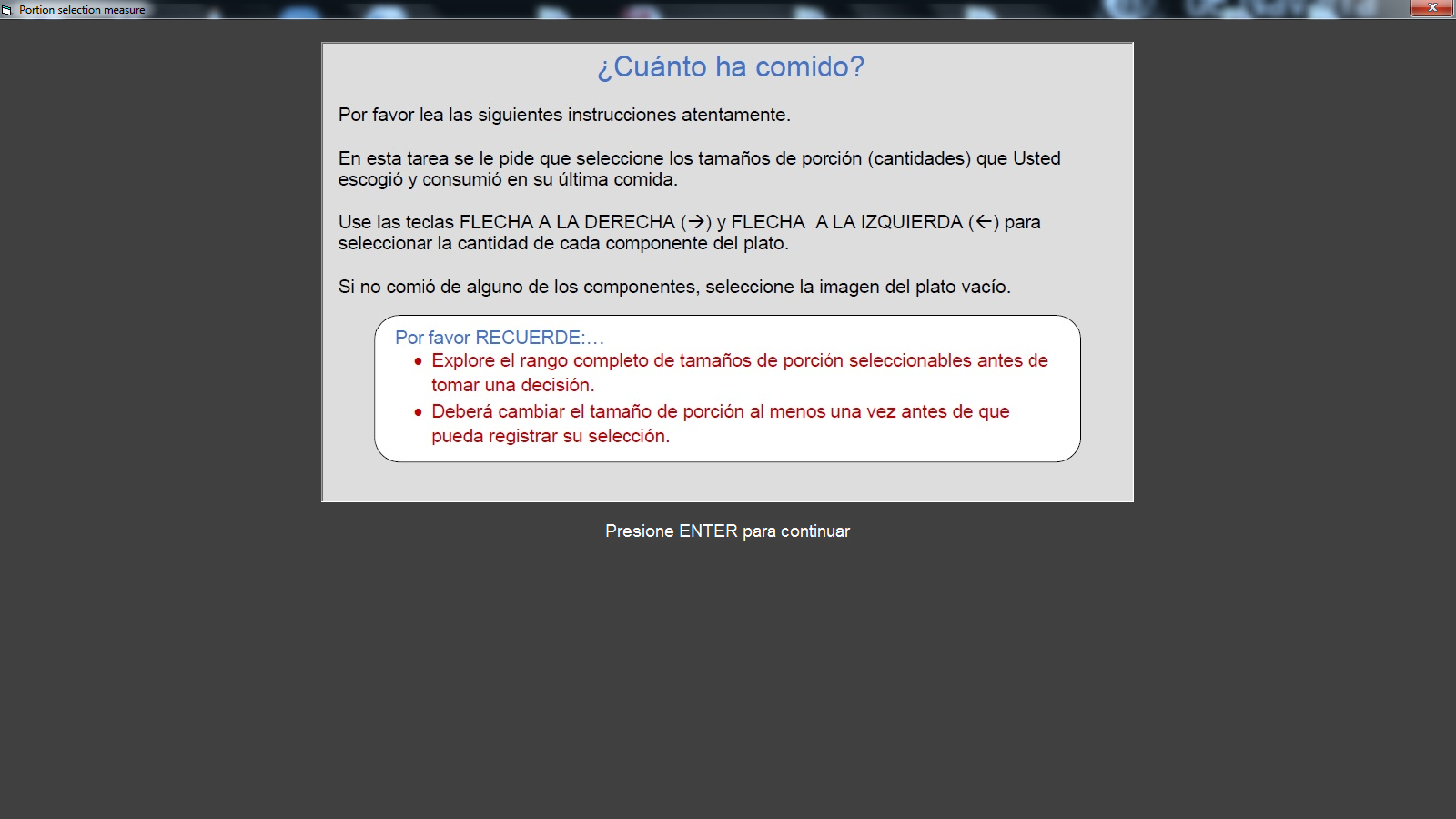


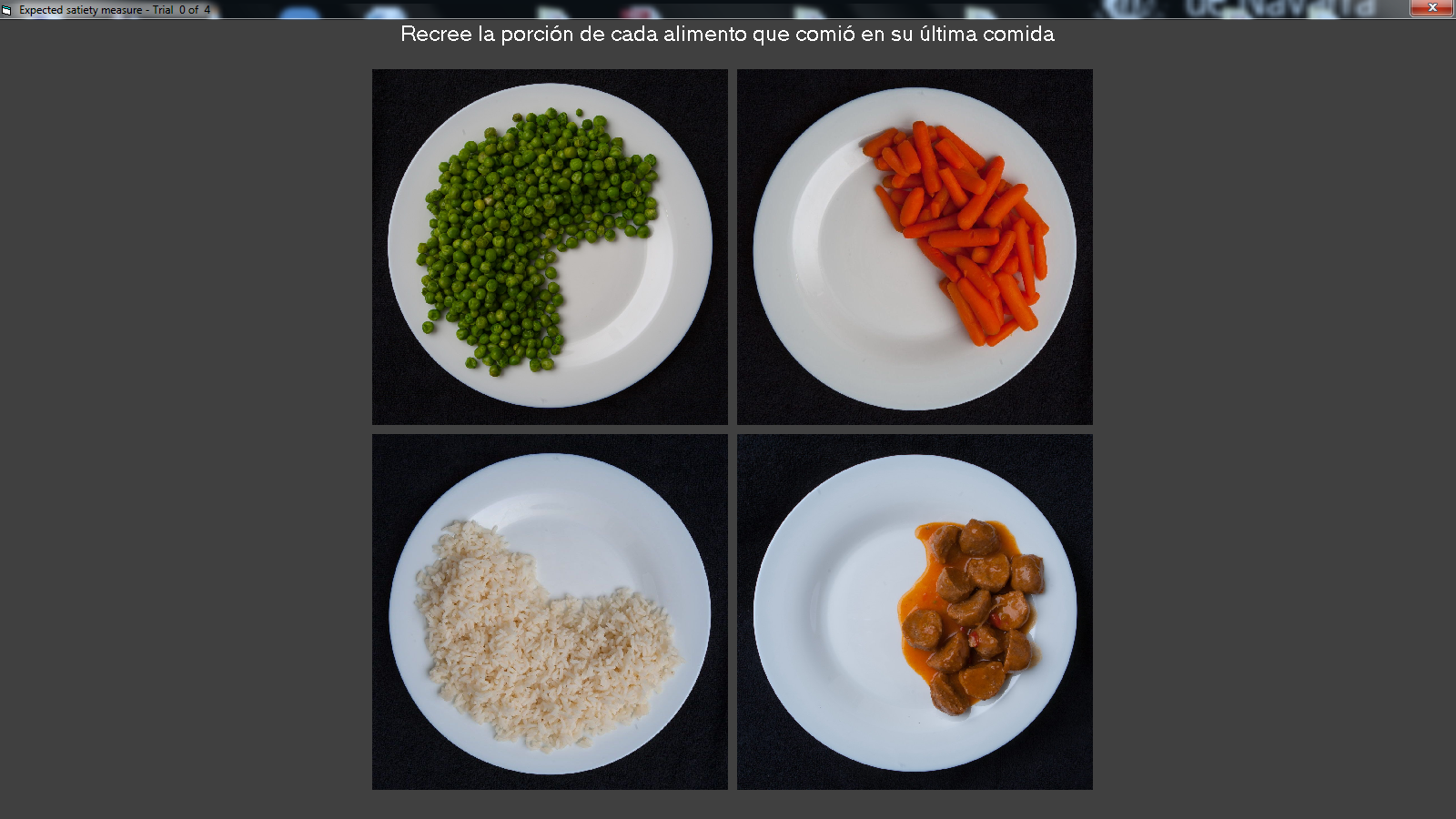


**Cited references**

1. Robinson E, Oldham M, Cuckson I, Brunstrom JM, Rogers PJ, Hardman CA. Visual exposure to large and small portion sizes and perceptions of portion size normality: Three experimental studies. *Appetite*. 2016;98:28-34. doi:10.1016/j.appet.2015.12.010

2. Forde CG, Almiron-Roig E, Brunstrom JM. Expected Satiety: Application to Weight Management and Understanding Energy Selection in Humans. *Curr Obes Rep*. 2015;4(1):131-140. doi:10.1007/s13679-015-0144-0

3. Hill AJ, Blundell JE. Nutrients and behaviour: Research strategies for the investigation of taste characteristics, food preferences, hunger sensations and eating patterns in man. *J Psychiatr Res*. 1982;17(2):203-212. doi:10.1016/0022-3956(82)90023-1

4. Flint A, Raben A, Blundell JE, Astrup A. Reproducibility, power and validity of visual analogue scales in assessment of appetite sensations in single test meal studies. *Int J Obes Relat Metab Disord*. 2000;24(1):38-48. http://www.ncbi.nlm.nih.gov/pubmed/10702749.

5. SWEET Project. Home Page - sweetproject.eu. https://sweetproject.eu/. Published 2019. Accessed October 5, 2021.

6. Almiron-Roig E, Domínguez A, Vaughan D, Solis-Trapala I, Jebb SA. Acceptability and potential effectiveness of commercial portion control tools amongst people with obesity. *Br J Nutr*. 2016;116(11):1974-1983. doi:10.1017/S0007114516004104

7. Fast LC, Harman JJ, Maertens JA, Burnette JL, Dreith F. Creating a measure of portion control self-efficacy. *Eat Behav*. 2015;16:23-30. doi:10.1016/j.eatbeh.2014.10.009

8. Salvucci DD, Goldberg JH. Identifying fixations and saccades in eye-tracking protocols. In: *Proceedings of the Symposium on Eye Tracking Research & Applications - ETRA ’00*. New York, New York, USA: ACM Press; 2000:71-78. doi:10.1145/355017.355028

9. Orquin JL, Ashby NJS, Clarke ADF. Areas of Interest as a Signal Detection Problem in Behavioral Eye-Tracking Research. *J Behav Decis Mak*. 2016;29(2-3):103-115. doi:10.1002/bdm.1867

10. Fuchs AF. The saccadic system. In: Bach-y-Rita P, Collins CC, Hyde JE, eds. *The Control of Eye Movements*. New York: Academic Press; 1971:343-362.

11. Ditchburn RW. The function of small saccades. *Vision Res*. 1980;20(3):271-272. doi:10.1016/0042-6989(80)90112-1

12. Duchowski AT. *Eye Tracking Methodology. Theory and Practice*. 3rd ed. Cham: Springer International Publishing; 2017. doi:10.1007/978-3-319-57883-5_1

13. Werthmann J, Roefs A, Nederkoorn C, Mogg K, Bradley BP, Jansen A. Can(not) Take my Eyes off it: Attention Bias for Food in Overweight Participants. *Heal Psychol*. 2011;30(5):561-569. doi:10.1037/a0024291

14. van der Laan LN, Papies EK, Hooge ITC, Smeets PAM. Goal-directed visual attention drives health goal priming: An eye-tracking experiment. *Heal Psychol*. 2017;36(1):82-90. doi:10.1037/hea0000410

15. Viera AJ, Garrett JM. Understanding interobserver agreement: The kappa statistic. *Fam Med*. 2005;37(5):360-363. http://www.ncbi.nlm.nih.gov/pubmed/15883903. Accessed June 12, 2018.

16. Stolarova M, Wolf C, Rinker T, Brielmann A. How to assess and compare inter-rater reliability, agreement and correlation of ratings: an exemplary analysis of mother-father and parent-teacher expressive vocabulary rating pairs. *Front Psychol*. 2014;5:509. doi:10.3389/fpsyg.2014.00509

17. Liao SC, Hunt EA, Chen W. Comparison between inter-rater reliability and inter-rater agreement in performance assessment. *Ann Acad Med Singapore*. 2010;39(8):613-618. http://www.ncbi.nlm.nih.gov/pubmed/20838702. Accessed June 29, 2018.

1. To select a coding method for the Lightworks protocol, a pilot test across a sub-sample of 6 recordings was performed during Feb 2018. As part of this pilot test, the same researcher coded video data using the *timestamp method* and the *frame counting method* for one of the videos. This revealed that the frame counting method was much more time efficient due to not having to write 2 timestamps (which contain 6 digits) for each entry.Therefore, this method was selected as the basis for the coding protocol. As this method can be prone to counting error, quality control measures were incorporated (e.g. double checking or double-counting for fixations longer than 25 frames). [↑](#footnote-ref-1)
2. Ref. ^1^: Hopkins WG (2009). Calculating the reliability intraclass correlation coefficient and its confidence limits (Excel spreadsheet). newstats.org/xICC.xls

   Ref. ^2^: Bartko, John J. 1966. “The Intraclass Correlation Coefficient as a Measure of Reliability.” *Psychological Reports* 19:3–11.

   Ref. ^3^ : McGraw, Kenneth O. and S. P. Wong. 1996. “Forming Inferences about Some Intraclass Correlation Coefficients.” *Psychological Methods* 1(1):30–46. [↑](#footnote-ref-2)
